# Exploratory Structural Equation Modeling: Practical Guidelines and Tutorial With a Convenient Online Tool for Mplus

**DOI:** 10.3389/fpsyt.2021.795672

**Published:** 2022-01-07

**Authors:** Llewellyn E. van Zyl, Peter M. ten Klooster

**Affiliations:** ^1^Department of Industrial Engineering, University of Eindhoven, Eindhoven, Netherlands; ^2^Optentia Research Focus Area, North-West University, Vanderbijlpark, South Africa; ^3^Department of Human Resource Management, University of Twente, Enschede, Netherlands; ^4^Institut für Psychologie, Goethe University, Frankfurt am Main, Germany; ^5^Psychology, Health and Technology, University of Twente, Enschede, Netherlands

**Keywords:** exploratory structural equation modeling, psychometrics, factor analysis, mental health continuum, statistical tutorials

## Abstract

Critics of positive psychology have questioned the validity of positive psychological assessment measures (PPAMs), which negatively affects the credibility and public perception of the discipline. Psychometric evaluations of PPAMs have shown that various instruments produce inconsistent factor structures between groups/contexts/times frames, that their predictive validity is questionable, and that popular PPAMs are culturally biased. Further, it would seem positive psychological researchers prioritize date-model-fit over measurement quality. To address these analytical challenges, more innovative and robust approaches toward the validation and evaluation of PPAMs are required to enhance the discipline's credibility and to advance positive psychological science. Exploratory Structural Equation Modeling (ESEM) has recently emerged as a promising alternative to overcome *some* of these challenges by incorporating the best elements from exploratory- and confirmatory factor analyses. ESEM is still a relatively novel approach, and estimating these models in statistical software packages can be complex and tedious. Therefore, the purpose of this paper is to provide novice researchers with a practical tutorial on how to estimate ESEM with a convenient online tool for Mplus. Specifically, we aim to demonstrate the use of ESEM through an illustrative example by using a popular positive psychological instrument: the *Mental Health Continuum-SF*. By using the MHC-SF as an example, we aim to provide (a) a brief overview of ESEM (and different ESEM models/approaches), (b) guidelines for novice researchers on how to estimate, compare, report, and interpret ESEM, and (c) a step-by-step tutorial on how to run ESEM analyses in Mplus with the De Beer and Van Zy ESEM syntax generator. The results of this study highlight the value of ESEM, over and above that of traditional confirmatory factor analytical approaches. The results also have practical implications for measuring mental health with the MHC-SF, illustrating that a bifactor ESEM Model fits the data significantly better than any other theoretical model.

## Introduction

Positive psychology emerged in the late 1990s to counterbalance the dominating psychopathological focus of the time (1). In the 22 years since its inception, positive psychology's strive to apply the scientific method to investigate the positive states, -traits, and -behaviors that enhance mental health, has spawned a magnitude of new theories, models and constructs (2, 3). The growth of the discipline resulted in a rapid rise in the development and use of “positive psychological assessment measures” (PPAMs) aimed at measuring these positive psychological constructs validly and reliably (4). However, critics of positive psychology have questioned the validity of PPAMs (5–8). Psychometric evaluations of PPAMs have shown that various instruments produce inconsistent factor structures, that reliability estimates vary significantly between groups/contexts/times frames, that the predictive validity is questionable and that popular PPAMs are culturally biased [cf. (9–11)]. Although these challenges apply to all self-report psychometric instruments aimed at measuring psychological phenomena, it is particularly damaging to the discipline as it fuels current scientific critiques of positive psychology [cf. (12, 13)]. These critiques, in turn, negatively affect the credibility and public perception of the discipline.

A typical example of these challenges can be seen with the *Mental Health Continuum-Short Form* [MHC-SF; (14)]. The MHC-SF is one of the most popular PPAMs aimed at measuring mental health, has shown to produce various factorial models ranging from a correlated three first-order factorial model (comprised of emotional-, psychological-, and social well-being) through to various types of bifactor models with varying ranges of reliability (15–18). Zemojtel-Piotrowska et al. (18) also showed that the MHC-SF is not equivalent between cultures and required various modifications of the factorial model to ensure that partial invariance could be established. Further, the item relating to “positive relationships” on the psychological well-being sub-scale, is strongly related to the social well-being subscale and has shown to load on both constructs in several contexts [cf. (18)]. In individualistic cultures, a clear distinction between these three factors is apparent, however in collectivistic cultures psychological- and social well-being seem to be tied more closely together (16, 18). Therefore, limiting the cross-cultural comparisons which could be made with the instrument. A final conceptual issue also pertains to how mental health is defined vs. how it's measured. Keyes (19) indicated that mental health lies on a continuum ranging from languishing to flourishing. However, the MHC-SF measures mental health as a function of “a dynamic interaction between three factors” classified into three categories (languishing, moderate mental health, and flourishing). When cross-loadings are constrained to zero in its estimation, no “dynamic interaction” between these factors can be captured. Further, the categorization of mental health into categories is not aligned with the idea that mental health ranges on a continuum. Therefore, there is a disconnect between the conceptual formation of mental health as a continuum and the psychometric measurement (or estimation) thereof as categorical. Given that factors like mental health cannot directly be observed but only inferred through behavioral observation, Morin et al. (20) argued that the approach employed to (analytically) explore and validate instruments measuring these factors may be at the core of the issue.

Marsh et al. (21) argued that behavioral observation in psychological research usually takes the form of recorded responses to observed indicators (items on questionnaires), reflecting the overall, unobserved latent factor it is supposed to be measuring. Factor analysis was therefore developed to explore and represent these psychological constructs through constructing latent factors that are seen as the “underlying cause of these behaviors” [(20), p. 1,044]. Although a variety of multivariate factor analysis techniques exist to model and explore the factorial structures of constructs, psychological research has broadly adopted exploratory factor analyses (EFA) and confirmatory factor analysis (CFA) as its methods of choice (20–22).

EFA refers to a set of statistical techniques used to identify or uncover the smallest number of relevant dimensions needed to explain the covariation amongst a set of measured items or variables (23). In other words, EFA aims to identify common factors in data that explains the order and structure amongst measured items (21). EFAs allow for factors to be freely estimated by the available data, and cross-loadings are permitted to achieve a simple and interpretable factorial solution (23). EFAs are not without their criticisms and limitations. EFAs cannot incorporate or control for method effect (24). For example, when two relatively similarly worded items are present in a questionnaire, the covariance between these cannot entirely be explained only by their relationship with the latent construct; a residual correlation would need to be added. Further, within the EFA framework, scores produced by an instrument cannot directly be compared with scores produced by other groups or even over time (23). Direct comparisons are only possible if the item and factor loadings are precisely the same for both groups (which in practice is unlikely). This further implies that factorial equivalence or measurement invariance cannot be estimated or compared (25). A final major limitation is that EFA is data-driven, limiting its usefulness to applied researchers wanting to conduct more complex analysis (21, 26).

In contrast, CFA was developed by Joreskog (26) as a theory-driven approach whereby factor structures rely purely on an a priori specification of unique items onto their respective latent factors. In other words, CFAs aim to explore how well a predefined theoretical model “fits” the data that has been collected. Here, researchers formulate several clear hypotheses about the nature of a construct (e.g., how many factors it comprises of, whether factors are related or not, which items load onto which construct etc.) before data collection or analysis (23, 27). These assumptions are then tested against the data, and different or “alternative” theoretically informed models are sequentially computed and compared to determine which fits the data best. Within CFAs, items are forced to be only related to one specific latent factor, whereby loadings on other factors are constrained to be zero (28). Unlike EFAs, CFAs actively model and incorporate item uniqueness and correct for measurement error (20). Further, CFA models tend to produce more parsimonious models, where latent variables are easier to understand and interpret (29). CFAs have become more dominant in their use over the last three decades due to the advent of Structural Equation Modeling (SEM) and more powerful computers to process data (21, 28). This allows researchers to model complex data and construct more “accurate” models of behavior given real-world scenarios (21).

Although CFAs within the SEM framework are currently probably the most widely used method to examine the factorial structure of an instrument in psychological research (20, 29), it is not without its limitations. Conceptually, CFA models are often overly simplistic, restrictive and idealistic as it assumes “pure factors,” where items only load onto their a priori latent factors (i.e., cross-loadings are constrained to zero) (21, 28, 30). Given that most items on psychological measures tend to measure more than one conceptually related factor, some degree of construct-relevant association between items can and should be expected (31). This naturally leads to several significant, yet small cross-loading items on non-target factors (29). Forcing items to then only load on one a priori latent factor and constraining cross-loadings to zero leads to a more parsimonious model but artificially inflates the associations of items with factors (27, 28). This, in turn, would lead to inflated model fit statistics and inflated measurement quality indicators, which results in positively biased factor correlations; unless all non-target factor loadings are close to zero (21, 27). Simulation studies have shown that even small cross-loadings need to be considered to avoid inflated parameter estimates and biased results (22). Therefore, this positive bias, along with constraining loadings to zero, could undermine the discriminant validity of more restrictive CFA models, as correlations between factor indicators is forced to only go through their main factors (22, 24). In practice, this distorts how the interrelationships between the constructs are interpreted and, therefore, also their meaning (22).

Another issue relates to the goodness-of-fit indices [cf. (32)], which CFA models rely on. These fit indices are usually too restrictive when applied to multi-factor psychological instruments. Therefore, it is almost impossible to achieve “good” data-model fit without significant modifications to the factorial models (33). However, when looking at item level indicators and measures of reliability, these same models that produce “bad fit” can produce reasonable item loadings and high levels of reliability (33–35). Researchers then tend to incorporate more dubious exploratory, data-driven, approaches within the CFA framework to enhance data-model fit, such as correlating residual error terms on items, item parceling, HARKing, or constraining paths to be equal (21). Similarly, various studies have anecdotally shown discrepancies between the reported EFA and CFA results, which cannot solely be due to multiple cross-loadings that were not correctly modeled [cf. (29, 31)]. Therefore, traditional CFA approaches do not seem to fit psychological constructs that well. This poses several challenges for positive psychological research as this positive bias undermines support for (a) the multidimensional view of psychological constructs and instruments assuming to measure such, (b) the discriminant validity of PPAM, (c) the predictive validity of psychometric instruments based on high levels of multi-collinearity, and (d) the practical, diagnostic usefulness of an instrument (22).

More innovative and robust approaches to validating and evaluating psychometric instruments are required to address these analytical challenges. Applying more innovative and flexible approaches to evaluating PPAMs could enhance the discipline's credibility and advance positive psychological science. Recent developments in the field have started to use more flexible approaches to factor analysis such as Bayesian estimation [cf. (36) for a gentle introduction] and even incorporating EFA approaches into CFA models through SEM in order to capitalize upon the strengths of both (20). One of these promising alternatives to overcome the restrictions posed by the aforementioned analytical frameworks is Exploratory Structural Equation Modeling (ESEM) (30).

### Exploratory Structural Equation Modeling

ESEM was developed to incorporate the best elements of both CFAs [e.g., predictive relationships between factors (adjusted for measurement error), can produce method factors, correlated item uniqueness, estimate complex error variance structures, and produce bifactor models, estimate measurement invariance, and even be specified into auto-regressive models) and EFAs (e.g., allowing cross-loadings] into the traditional SEM framework (30). Therefore, ESEM provides a compromise between the mechanical iterative approach toward finding optimal factorial solutions through rotations within an EFA and the restrictive a priori theory-driven modeling approach employed within CFA measurement models (20).

Marsh et al. (22) stated that ESEM is fundamentally a confirmatory technique (although it can be used in an exploratory way), which through a target rotation, makes it possible to model data in a confirmatory way by allowing for the presence of cross-loadings between items. Although permitted, cross-loadings (non-target loadings) are constrained to be as close to zero as possible (30). Drawing from CFAs, within the ESEM framework, the researcher has more a priori control over the expected factor structure of an instrument. Further, how ESEM models identify mean structures is typically similar to traditional CFA models where item intercepts are estimated freely and latent factor means are constrained to zero (22, 30). Given that a CFA model is also nested within an ESEM model, both models can directly be compared through traditional model fit indices (22). When an ESEM solution fits the data better than a traditional CFA model, the estimated factor correlation is likely to be substantially less biased than in the CFA model (22).

ESEM incorporates more flexible EFA models into its model estimation by allowing items to cross-load on non-target factors. However, this means that the rotation method employed is critically important as the size and direction of the estimated factor correlations differ depending on the type of rotation (22). Rotation procedures are required for model identification but are employed to simplify the interpretability of the factors which ESEM/EFAs tend to estimate (37). The choice of rotation procedure directly affects the estimated factor correlations and cross-loadings (37). Xiao et al. (29) indicated that the three most popular rotation methods employed in ESEM are the (oblique) geomin- and target rotations, with orthogonal rotations being used for bifactor ESEM models. Asparouhov and Muthén (30) stated that geomin rotations, where correlations between factors are estimated and incorporated, generally perform well if the estimated model isn't too complex. On the other hand, target rotations allow ESEM model estimations to be used more confirmably but depend on the a priori assumptions made about how cross-loadings are specified (30). Target rotations do not require a researcher to specify “anchor items” with non-targeted factor loadings (22) and provide more control in specifying models (30). This implies that more complex models can be estimated when using target rotation and should be preferred (30). In bifactor ESEM models, the general (G) factor and the specific (S)factors need to be specified as totally independent from one another, and therefore the relationships between factors and variances shared need to be constrained through the orthogonal rotation (37).

ESEM poses several advantages over and above those of the traditional CFA and EFA approaches. Morin et al. (20) and Marsh et al. (22) argued that ESEM is more robust, rigorous, and flexible than most analytical approaches as (a) it can simultaneously estimate both CFA and EFA models, (b) it can estimate less restrictive measurement models that permit cross-loadings which can produce useful-fit indices and parameter estimates, (c) it usually fits the data significantly better than traditional CFA and EFA models, (d) latent factor correlations are less biased and are closer to the true associations and most importantly, (e) these models are usually also more in line with the theoretical conceptualization and considerations of the construct the instruments intend to measure. Van Zyl et al. (38) also showed that ESEM models could potentially compensate for wording effects and cross-cultural differences in the interpretation of items when comparing different national or cultural groups. It is, however, essential to note that ESEM does not necessarily increase the instrument's reliability and that, despite improving model-fit, researchers should always carefully inspect all item-level parameters. Further, various typical simple and more complex CFA model permutations can also be estimated within the ESEM framework, such as first-order-, hierarchical-, and bifactor models (39, 40). As such, ESEM could be used for several purposes such as scale construction (like traditional EFA), refinement and validation (like traditional CFAs), and replication (40).

Although there are several advantages of ESEM, it also has several limitations. Traditional (first-order) ESEM models cannot easily be used in more complex, predictive, or hierarchical models (20, 41). For example, Morin et al. (24) argued that the bootstrapped confidence intervals required to provide support for the indirect effect of a mediator on the relationship between an exogenous and endogenous factor cannot be generated with ESEM models. In other words, one would for instance, not be able to determine how mental health indirectly affects the person-environment-performance relationship. Normal first-order ESEM factors can also not meaningfully be used as indicators for higher-order factors which limits its use in, for example, full latent growth curve models (42). Morin et al. (20) argued that to do so, the higher-order factorial model should be constructed based on the correlations of the first-order factors. However, this provides nothing more than just a simple expression of these inter-factor correlations that do not accurately represent the hierarchical nature of a multidimensional construct. Current estimation procedures for ESEM models, also do not support multilevel- or mixture modeling (39) nor mixture models (42), thus limiting its use in, for example, daily-diary intervention studies. According to Marsh et al. (39) it is also not presently possible to accurately estimate partial factorial invariance. Morin et al. (42) also indicate that latent means can't be constrained in multi-group models, and therefore comparisons between (for example) genders/ages/cultures on mental health is not possible. Marsh et al. (39) also mentioned that within ESEM, multiple sets of (unrelated) factors cannot be estimated simultaneously, as permitting for cross-loadings between these factors (e.g., mental health vs. performance) would undermine the theoretical foundation of both factors. Further, full ESEM models may lack parsimony and that the popular (dubious) approaches to circumvent such used within CFAs (such as item parceling, or using manifest scale scores) could not be used to ensure convergence (22, 39). Another limitation is that using ESEM models within structural models may present convergence and estimation problems (24). For the applied researcher, ESEM models may therefore not be useful above and beyond exploring the factorial validity of an instrument.

To address these issues, and circumvent the limitations of ESEM, *set-ESEM* (39), and *ESEM-within-CFA* (22, 24) was developed. *set-ESEM* allows for the modeling of two or more distinct “sets” of constructs within a single (ESEM) model, where cross-loadings between items are allowed for (first-order) factors that are related to the same construct (or set) but constrained to zero for constructs of different sets (like within a traditional CFA model) (39). These sets could reflect the same construct at different time stamps in longitudinal models or different constructs measured simultaneously within cross-sectional data. For example, if common mental health problems [stress, depression, and anxiety as measured by the DASS-21: (43)] and mental health [emotional-, psychological-, and social well-being as measured by the MHC-SF: (14)] are estimated within set-ESEM, then both “sets” of factors would be modeled simultaneously. Here, the first-order latent factors would be permitted to covary, and cross-loadings between the DASS-21 factors and cross-loadings between the MHC-SF factors would be permitted. However, unlike within a full ESEM model, items from the DASS-21, would not be permitted to cross-load with the MHC-SF and vice versa. Set-ESEM allows for the simultaneous estimation of multiple constructs and finds an optimal balance between CFAs and ESEMs in respect of parsimony, data-model fit, rigor, and well-defined factor estimation (39). set-ESEM therefore also maintains the structural (theoretical) integrity of each set of ESEM models whilst allowing for more flexibility in estimation. Set-ESEM is, however, still a relatively new approach within the ESEM lexicon, and its full practical usefulness needs to be explored. Its therefore beyond the scope of this tutorial to fully reflect upon the technique [interested readers are referred to (39) for a non-technical overview of set-ESEM].

*ESEM-within-CFA*, on the other hand, assumes that the resulting measurement structure of an ESEM factor model would remain stable when transformed into a CFA model (39). Within this framework, a first-order ESEM model is re-expressed within a CFA framework by using the (unstandardized) factor loadings of the ESEM model as starting values (42), and factor variances are freely estimated (44). The original ESEM solution and the respecified ESEM-within-CFA solution should produce precisely the same chi-square, degrees of freedom, model-fit statistics, and parameter estimates (standard errors may be slightly inflated, though) (42). By expressing an ESEM model within the ESEM-within-CFA framework, more “traditional” models and analyses can be conducted. For example, hierarchical or “second-order factor” ESEM models of mental health could be constructed where mental health is a function of these three first-order ESEM factors. Morin et al. (24) for example showed that partial mediation could be estimated with the ESEM-within-CFA model and that bias-corrected bootstrapped confidence intervals could be produced. Morin et al. (42) also showed that the ESEM-within-CFA solution could be used to show how factors change over time, longitudinal mediation and could even be used to estimate latent change score models. Further, Howard et al. (45) also showed that an ESEM-within-CFA model could be used in normal structural models to establish the relationships between factors. Therefore, the ESEM-within-CFA framework makes it possible for applied researchers to readily use ESEM models for more complex research questions.

Given these advantages, it's therefore not surprising that the use of ESEM is gaining popularity within the positive psychological sciences (46–49). However, there are several challenges its use poses for positive psychological researchers:

1. The ESEM approach is complex, and its usefulness is challenging to articulate to applied scientists.
2. Given that it is a relatively recent development applied scientists may find it difficult to understand when, where, why, and how to use ESEM and find it challenging to understand what the results mean and what to report. To the best of our knowledge, no best practice guidelines for estimating and reporting ESEM are easily accessible to the average researcher.
3. Finally, there are currently only two software packages that can estimate ESEM models: R Studio and Mplus (50). Mplus is currently the only software package that fully integrates ESEM, whereas R currently only provides partial implementation (37). Estimating ESEM models in either software package requires complex code or syntaxes to run. Further, especially estimating ESEM-within-CFA models is not only complex, but extremely tedious and time-consuming.

These three challenges may significantly hamper researchers to adopt ESEM as an alternative to traditional EFA and CFA modeling strategies. Specifically, the perceived complexity and unfamiliarity with the approach and its estimation procedure may reduce the probability of less experienced researchers exploring or using these alternative factor analytical techniques. Therefore, the purpose of this paper is to provide novice researchers with a practical tutorial on how to apply ESEM with an innovative tool for Mplus. Specifically, we aim to demonstrate the use of ESEM through an illustrative example by using a popular positive psychological instrument: the *Mental Health Continuum-Short Form* [MHC-SF: (14)]. By using the MHC-SF as an example, we aim to provide (a) a brief overview of ESEM (and different ESEM models), (b) guidelines for novice researchers on how to estimate, compare, report and interpret ESEM models, and (c) a step-by-step tutorial on how to run ESEM analyses in Mplus with an easy to use online tool for syntax generation.

### The Mental Health Continuum: An ESEM Perspective

Mental health is a foundational component in the positive psychological lexicon and of keen interest to researchers and practitioners alike (1, 51, 52). Therefore, mental health is a popular and familiar framework that applied positive psychological researchers can relate to and thus an interesting concept to use to illustrate ESEM.

Mental health is defined by the World Health Organization [(53), p. 2] as “*a state of wellbeing in which the individual realizes his or her own abilities, can cope with the normal stresses of life, can work productively and fruitfully, and is able to make a contribution to his or her community*.” This definition implies that mental health is a function of (a) overall well-being, (b) effective psychological functioning, and (c) successful integration in and contributions to society (54). These elements form the foundation for a popular approach to mental health in positive psychology called “The Mental Health Continuum” (14). Developed by Keyes (55), this approach defines mental health as “a syndrome of symptoms of positive feelings and positive functioning in life” (p. 207) where an individual is “free of psychopathology and flourishing with high levels of emotional-, psychological-, and social well-being” [(14), p. 539]. From this perspective, Keyes et al. (56) argued that mental health is more than just being free from psychopathology and is an active function of *feeling good* (i.e., *emotional well-being (EWB)*: pursuing pleasure, avoiding pain and experiencing affect balance), *functioning well* (i.e., *psychological well-being (PWB)*: having the capabilities to manage life's challenges and realize one's potential effectively), and *fitting in* (i.e., *social well-being (SWB)*: the extent toward which one optimally functions in, feels accepted by and contributes to their community). Keyes (55) argued that mental health could be described on a continuum between languishing on the lower end and flourishing at the top end of the spectrum. Further, Westerhof and Keyes (54) argued that mental health and mental illness are on separate, yet related continuums where one could (in theory) be both flourishing yet suffering from mental illness. Mental health can therefore be seen as a complete state of well-being whereby individuals have balanced positive/negative experiences, are free to realize their full potential, can play to their strengths to manage daily hassles and can actively contribute to the communities they are embedded in (57, 58).

This mental health approach and definition served as the basis for the *Mental Health Continuum-Short Form* [MHC-SF; (59)], a popular 14-item self-report measure that aims to assess individuals' overall level of emotional-, psychological-, and social well-being. The instrument assesses mental health as a continuum that ranges from flourishing and moderately mentally healthy to languishing (14). Flourishing is constituted by elevated emotional, psychological, and social functioning levels and languishing by low self-reports on these factors. Mental health is represented by a higher-order factorial model, which assumes that the three first-order factors completely mediate the association between the 14 items and the higher-order well-being factor. This implies that the higher-order factor does not explain any unique variance over and above what is already explained by EWB, PWB, and SWB. Therefore, these three first-order factors confound the variance explained by the higher-order factor, and the variance is uniquely attributable to each of the three first-order factors (20). This factorial model relies heavily on implicit restrictive proportionality constraints whereby the ratio of item variance explained by the first- and higher-order factors is the same for all items associated with the single first-order factor (60). Despite such, this higher-order factorial model is still very prevalent in the literature. The MHC-SF has been adapted, translated, and validated in over 50 countries, thus providing considerable support for its utility and validity (56). Given the instrument's popularity, it has also been subjected to a wide array of structural validity studies, where various factorial permutations have been investigated, ranging from traditional CFAs to more complex ESEMs (58).

From a *traditional CFA perspective*, the MHC-SF has been estimated as a:

1. **Strict uni-dimensional model**, where all items directly load on a single first-order factor model, and more recently:
2. **Three-factor first-order model**, where EWB, PWB, and SWB are estimated as distinct and related (correlated) first-order factors.
3. **Higher-order (second-order) model**, where mental health is estimated as a single, second-order factorial model comprised out of three first-order factors (EWB, SWB, PWB). This CFA model is mathematically equivalent to the previously mentioned model for the MHC-SF.
4. **Bifactor model**, where mental health is seen as a general factor, that is district from the three independent specific factors. A bifactor model provides an alternative to traditional higher-order factorial models as items can simultaneously reflect an overall or “general” factor of mental health (G-factor) and three specific factors (S-factors) reflecting the unique variance shared amongst the items forming each of the three subscales that the G-factor does not explain. Therefore, the G-factor reflects the variance shared by all indicators in the model, where the S-factors represent the shared variance among all the indicators of a specific subscale that's not accounted for by the G-factor. These factors are specified as orthogonal (i.e., being unrelated to each other and therefore unique). This approach aids in solving issues related to high factor correlations and acts as a means to determine the unique contribution of the G-factor and the S-factors to predictive outcomes. Jovanovic (15) found that mental health is better represented by a bifactor model, rather than any of the other theoretical CFA permutations.

More recently, the MHC-SF has also been explored through a variety of ESEM approaches. Joshanloo and Jovanovic (25) argued that the traditional CFA approaches don't adequately represent the multi-dimensionality of the MHC-SF, and that ESEM results in better model fit, provides more accurate parameter estimates and projects more realistic inter-factor correlations. Further, ESEM structures are more closely aligned to the original theoretical conceptualization of mental health as laying on a continuum, where flourishing results from an active interaction of EWB, PWB, and SWB (47). Empirically, employing a CFA approach undermines the continuum conceptualization as it forces factors to be “categorical,” instead of allowing for the dynamic interaction required to theoretically constitute a “continuum.” Following the a priori factorial structure of the MHC-SF, Lamborn et al. (61), Joshanloo and Jovanovic (25), Joshanloo and Lamers (62), and others indicated that the MHC-SF is better represented by one of the following ESEM models where cross-loadings were permitted but targeted to be close to zero:

1. **Three-factor first-order ESEM model**, where EWB, PWB, and SWB are estimated as distinct and related first order factors. However, within hierarchically organized constructs such as mental health, these first-order ESEM models are more likely to ignore the presence of hierarchical superior constructs as this would instead be expressed through hyper-inflated cross-loadings (42). Hierarchical ESEM models could therefore be estimated.
2. **Higher-order ESEM model (H-ESEM)**, where mental health is estimated (*via* the ESEM-within-CFA framework) as a single, second-order factorial model comprised out of three first-order factors (EWB, SWB, PWB).
3. **Bifactor ESEM model**, where mental health is seen as a general factor, that is district from the three independent specific factors.
4. **ESEM-within-CFA model**, could thus also be specified where the three-factor first-order ESEM model is re-expressed within a CFA framework using the starting values of the original three-factor first-order ESEM. This approach allows the ESEM model to be used in more complex analyses. However, it has not yet been employed with the MHC-SF but will be demonstrated later in this tutorial.

## The Present Study

This paper aims to provide an illustrative tutorial on the specification, comparison, reporting, and interpretation of ESEM models in Mplus with the aid of De Beer and Van Zyl's (63) ESEM Code Generator. The ESEM Code Generator assists with generating syntaxes for Mplus estimation based on the basic factor structure of an instrument, limiting the potential for inadvertent errors in manual model specification.

Specifically, this aim translates into two objectives:

1. To provide general guidelines to consider when estimating, interpreting, comparing and reporting ESEM models.
2. To provide a step-by-step guide for estimating ESEM models in Mplus with the ESEM Code Generator (63) and comparing ESEM models with traditional CFA models.

For illustrative purposes, data obtained by the LISS Open Data Project will be used to explore the factor structure of the MHC-SF. Both traditional CFA and ESEM models will be estimated and compared. For the sake of familiarity, the illustration will follow the format of a traditional paper's methods and results section accompanied by ESEM Code Generator and syntax screenshots and guidelines.

## Materials and Methods

### Research Design

The study draws on data from the LISS internet panel of the CentERdata programme (https://www.dataarchive.lissdata.nl). The LISS panel is a functional element of the Measurement and Experimentation in the Social Sciences (MESS) project managed by CentERdata in Tilburg, the Netherlands. The panel gathers longitudinal data from a representative sample of 5,000 random households based on the population register by Statistics Netherlands. For this study, the first measurement of the 2007 Dataset on Mental Health was used (*n* = 1,806). Overall, 2,293 individuals were invited to participate in the study with a 78.7% response rate.

### Participants

Data were gathered from a random, representative sample of 1,806 respondents from the Netherlands. Data were screened for response quality which led to the removal of two records from the final data set (64).

The final sample consisted of 1,804 participants (cf. Table 1). The majority of the participants were married (53.9%) Dutch females (50.7%) who were 65 years or older (21.4%). Most had at least a higher vocational level of education or a university degree (29.7%) and lived in self-owned housing (68.2%).

**Table 1:** *Demographic characteristics* of participants (*N* = 1,804).

**Characteristics**	**Category**	**Frequency (*f*)**	**Percentage (%)**
Gender	Male	890	49.3
	Female	914	50.7
Age	15–24 years	194	10.8
	25–34 years	362	20
	35–44 years	277	15.4
	45–54 years	279	15.5
	55–64 years	306	17
	65 years and older	386	21.4
Marital status	Married	973	53.9
	Separated	5	0.3
	Divorced	172	9.5
	Widow or widower	103	5.7
	Never been married	551	30.5
Level of education	Primary school	80	4.4
	Vmbo (intermediate secondary education, US: junior high school)	469	26
	Havo/vwo (higher secondary education/preparatory university education, US: senior high school)	210	11.7
	Mbo (intermediate vocational education, US: junior college)	392	21.7
	Hbo (higher vocational education, US: college)	392	21.7
	Wo (university)	144	8
	Other	72	4
	Not yet completed any education	44	2.4
	Not yet started any education	1	0.1
Type of living arrangement	Self-owned dwelling	1,229	68.2
	Rental dwelling	562	31.1
	Cost-free dwelling	13	0.7

### Measures

The *Dutch version* of the *Mental Health Continuum-Short Form* [MHC-SF; (55)] was used to measure overall mental health and its three components. The instrument consists of 14 self-report items that are rated on a 6-point Likert scale ranging from 1 (“Never”) to 6 (“Every Day”). The instrument requests participants to reflect on the past month and indicate to what extent they experienced the three components of mental health: (a) emotional well-being (e.g., “happy”), (b) psychological well-being (e.g., “That you liked most parts of your personality”) and social well-being (e.g., “That you had something important to contribute to society”). The instrument showed to be highly reliable in the Dutch context, with Cronbach's alphas ranging from 0.89 to 0.93 on the different subscales (38).

The *Dutch version* of the *Brief Symptom Inventory* [BSI: (65)] was used to measure mental illness. The scale consists of 90 items which measure nine common mental illnesses (Somatization, Obsession-Compulsion. Interpersonal Sensitivity, Depression, Anxiety, Hostility, Phobic Anxiety, Paranoid Ideation, and Psychoticism) *via* a five-point Likert type scale ranging from 0 (“Not At All”) to 4 (“Extremely”). Each dimension is measured by 10 items. Example questions are: “During the past 7 days, how much were you distressed by feeling easily annoyed or irritated?” and “During the past 7 days, how much were you distressed by feeling lonely?”. The overall scale and its sub-dimensions showed to be reliable, with Cronbach Alphas ranging from 0.70 to 0.95 (54). Within the LISS Dataset, only total (factor) scores for each specific factor are provided due to copyright restrictions.

### Data Availability and Syntaxes

The data and syntaxes used for this tutorial are available as Supplementary Material to this manuscript. The Supplementary Material contains: (a) the original dataset in SPSS version 27 format (Mplus.sav), (b) the cleaned dataset used in Mplus (mplus.txt), (c) the ESEM syntaxes generated by the De Beer and Van Zyl (63) ESEM code generator for Mplus, and (d) the syntaxes used to estimate the CFA factor models.

### Guidelines for ESEM Estimation

To estimate, compare and report on ESEM, general guidelines were developed based on the best-practices for CFAs (cf. Table A1 for a summary). These general guidelines divide the procedure into three phases: (a) The Planning Phase, (b) the Data Preparation Phase, and (c) The Data Analysis and Reporting Phase.

#### The Planning Phase

*First, a clear explanation of the instrument, its factorial structure and possible alternative factorial models of such should be described based on theory*. When validating a psychometric instrument, clear, theory-informed hypotheses about the instrument's factorial structure or “nature” should be provided. Given that a CFA structure is nested within ESEM, a description of the traditional CFA models' original or expected factorial structure is required. As such, alternative, theory-informed, factorial permutations of the instrument should also be discussed and briefly described. These models may reflect different theoretical propositions underpinning the instrument or contradictions found in previous research (66). If the constructs within a CFA model are expected to be conceptually related, then there is also an expectation that an ESEM model would fit the data. Therefore, both the CFA and ESEM models of the different theoretical models need to be tested against the data to find the best data-model fit. If a global factor can be expected, then bifactor CFA and bifactor ESEM models should be described and later tested (37). In studies where the focus is on establishing relationships between factors or growth over time, the relationships between factors should be clearly described and supported by the literature.

*Second, the required sample size should be planned for* (67–69). Given that a “relatively large number of parameters need to be estimated in ESEM, smaller sample sizes could lead to decreased precision in model estimation” [(42), p. 3] and present problems with convergence (30), researchers should plan for an appropriate sample size beforehand. Given that CFA models are nested within ESEM models, traditional approaches for sample size estimation for SEM models could also be appropriate to help control for possible convergence problems later. Many different suggestions and rules of thumb for sample size planning have been proposed for SEM and CFA in the literature which researchers could consider [cf. (70–73)]. Wolff et al. [(73), p. 3], did however, suggested three more advanced approaches to estimate the sample size requirements for SEM, whereby the required sample size is estimation based on: (a) the non-centrality parameter (i.e., based on the amount of model misspecification) (74), (b) the model's potential to obtain an acceptable RMSEA value (71), or (c) Monte Carlo simulations (75). The latter, being the most preferred approach [cf. (73) for an easy tutorial with Mplus code for running Monte Carlo simulations to estimate sample size]. However, the actual necessary sample size depends to a large extent on the researcher's goals, and it is up to the researcher to decide which approach to employ.

#### Data Preparation Phase

Third, the *dataset needs to be screened, cleaned and prepared for analysis*. The dataset needs to be screened for outliers, missing values and an appropriate missing values strategy (e.g., multiple imputations, Full Information Maximum Likelihood estimation, sensitivity analysis, expectation-maximization, etc.) employed before or during analyses. The choice of strategy should be reported and justified. To determine potential multivariate outliers, the Mahalanobis distance estimation method could be used (*p* < 0.01) (76). Outliers and extreme values might need to be removed from the dataset as these could affect model fit and measurement quality (76, 77). Further, data quality checks should be implemented [cf. (64) for a review on possible strategies].

*Fourth, the most appropriate software, estimation method, rotation and procedure for the analysis should be decided and reported*. For this illustration Mplus 8.6 (50) will be used. Once the software package has been selected, an appropriate estimation method should be decided. By default, Mplus employs the Maximum Likelihood (ML) estimation method. Morin (37) suggests the use of robust ML (MLR) from the start as it compensates for issues pertaining to multivariate normality, however, additional steps should then be implemented for statistical model comparison as chi-squares cannot directly be compared for these estimation methods (e.g., the Satorra-Bentler scaled chi-square difference test which is also implemented in Mplus with the DIFFTEST command for models with different estimators). For bifactor ESEM models with continuous indicators, the MLR estimator is appropriate; for models comprised of ordinal indicators, WLSMV should be used. Once the estimator has been chosen, the most appropriate rotation method for ESEM should be decided and reported. Three rotations are to be considered depending on the purpose of the study: (a) Geomin rotations (with an epsilon value of 0.50) for more exploratory approaches and to maximally reduce factor correlations, (b) Target rotations for confirmatory approaches, or (c) (Target) Orthogonal rotations are used for bifactor ESEM modeling (37). Finally, the entire competing measurement modeling strategy to be employed should be described.

#### Data Analysis and Reporting Phase

*Fifth, the most appropriate goodness-of-fit indices and indicators of measurement quality should be determined and reported*. To determine the best fitting model for the data, both guidelines for goodness-of-fit indices as well as indicators of measurement quality need to be employed. Measurement models need to show both good data-model-fit and have high levels of measurement quality to be retained (33). To determine goodness-of-fit, Hu and Bentler's (55) proposed a number of general fit indices with suggested cut-off scores which are summarized in Table 2. It is, however, essential to note that each suggested model fit indicator is subjected to its own limitations and the use thereof needs to be justified. As such, multiple indicators of fit should be used to decide upon the best fitting model for the data (33). The CFI, TLI, and RMSEA should always be reported and used as the primary criterion for both establishing model fit and to discriminate between models. However, recent research has shown that SRMR outperforms RMSEA when the data that is modeled is categorical in nature (78).

**Table 2:** Model fit statistics.

**Fit indices**	**Cut-off criterion**	**Sensitive to *N***	**Penalty for model complexity**
***Absolute fit indices***			
Chi-Square (χ^2^)	• Lowest comparative value between measurement models	Yes	No
	• Non-Significant Chi-Square (*p* > 0.01)		
	• Significant difference in Chi-Square between Models		
	• For Model Comparison: Retain Model with Lowest Chi-Square		
***Approximate fit indices***			
Root-Means-Square Error of Approximation (RMSEA)	• 0.06–0.08 (Marginally Acceptable); 0.01–0.05 (Excellent)	No	Yes
	• Not-significant (*p* > 0.01)		
	• 90% Confidence Interval Range should not include Zero		
	• 90% Confidence Interval Range should not overlap between models		
	• For model comparison: Retain Model where ΔRMSEA ≤ 0.015		
Standardized Root Mean Square Residual (SRMR)	• 0.06 to 0.08 (Marginally Acceptable); 0.01–0.05 (Excellent)	Yes	No
	• For model comparison: Retain Model where ΔSRMR ≤ 0.015		
***Incremental fit indices***			
Comparative Fit Index (CFI)	• 0.90 to 0.95 (Marginally Acceptable Fit); 0.96 to 0.99 (Excellent)	No	No
	• For model comparison: Retain Model with Highest CFI value (ΔCFI > 0.01)		
Tucker-Lewis Index (TLI)	• 0.90 to 0.95 (Marginally Acceptable Fit); 0.96 to 0.99 (Excellent)	No	Yes
	• For model comparison: Retain Model with Highest TLI value (ΔTLI > 0.01)		
Akaike Information Criterion (AIC)	• Lowest value in comparative measurement models	Yes	Yes
Consistent AIC (CAIC; calculated as BIC + free parameters	• Lowest value in comparative measurement models	Yes	Yes
Bayes Information Criterion (BIC)	• Lowest value in comparative measurement models	Yes	Yes
Sample-Size Adjusted BIC (aBIC)	• Lowest value in comparative measurement models	Yes	Yes

After model-fit is established, measurement quality needs to be assessed. Researchers should decide, a priori, on indicators of measurement quality that can range from inspecting the standardized factor loadings (e.g., λ > 0.35), the item uniqueness (e.g., residual error variances >0.10 but <0.90), levels of tolerance for cross-loadings, and overall *R*^2^ for each item. However, the results should be considered in the context of the study and what they might mean or indicate without being unnecessarily rigid about minor deviations from the aforementioned rules of thumb.

Sixth, *estimate- and report the model fit indicators for various competing CFA models*. In this step, different theoretical factorial models should be estimated and their model fit statistics reported. For the MHC-SF, theory indicates that four types of CFA models are possible: A single factor model, three-factor first-order model, a second-order model and a bifactor model. Here measured items are used as observed indicators for latent factors. For the CFA models, items should only be allowed to load on their a priori theoretical factors and cross-loadings of items should not be permitted. For bifactor models (B-CFA) an orthogonal target rotation should be employed and specified in Mplus under the Analysis Command: ROTATION = TARGET (ORTHOGONAL). Here, a general factor for overall Mental Health (G-factor) should be specified, accompanied by emotional well-being, psychological well-being, and social well-being as specific factors (S-factors). For the G-factor, all observed indicators (measured items) need to be specified to load onto this single factor. For the three S-factors, items related need to be specified to load onto their a priori factorial structures. The orthogonal targeted rotation forces all factors to be uncorrelated. In other words, all covariances between the specific factors and general factor are constrained to be zero. This can also be manually specified in Mplus (e.g., EWB WITH PWB@0;). Further, any potential modifications made to the CFA models to enhance model fit should be reported and justified in text. Only modifications with a strong theoretically informed reason should be permitted. All model fit statistics (mentioned in Table 2) for the various models should be tabulated and reported.

*Seventh, estimate and report the model fit indicators for various competing ESEM models*. Like the previous step, various theoretically informed ESEM models need to be estimated and their model fit statistics reported. For the MHC-SF, theory indicates that three types of ESEM models are possible: a three first-order-, a second-order-, and a bifactor ESEM model. Additionally, an ESEM-within-CFA model could also be estimated based on the first-order ESEM model if more complex analyses are later required. This should be done in Mplus, and a target rotation should be employed. Unlike within the CFA models, cross-loadings between items and non-target factors are permitted; however, these should be constrained to be as close to zero as possible (79) (in Mplus, this is indicated by ~0; after the specific cross-loadings). Items that load onto their a priori theoretical latent factor should not be constrained. For the bifactor ESEM (B-ESEM) model, a similar approach as mentioned for the B-CFA model should be employed where the MHC-SF is comprised of a single G-Factor and three S-Factors. However, unlike in the B-CFA model, cross-loadings on non-target S-factors are permitted but targeted to be as close to zero as possible. The code for all the ESEM models can be generated with the De Beer and Van Zyl (63) ESEM code Generator for Mplus. This will be explained in the next section. All observed model fit statistics (mentioned in Table 2) for the various models should be tabulated and reported. It is suggested that both the CFA and ESEM results be reported in the same table, making model comparisons easier to read.

*Eight, to determine the best-fitting model for the data, the competing CFA and ESEM models need to be compared*. In this step, the results for both Steps 6 and 7 are compared, based on the criteria specified in Table 2, to determine the best-fitting model for the data. Given that the CFA models are embedded within the ESEM models, direct comparisons on model fit can be made. Only models that meet both the measurement quality and goodness-of-fit criteria should be retained for further analyses. Models with the lowest AIC, BIC and aBIC values show better fit and should be favored. For competing nested models, a model shows better fit if both the chi-square difference test between models is significant (*p* < 0.05) and changes in RMSEA/SRMR and TLI/CFI exceed 0.015 and 0.01, respectively (80, 81). It should, however, be noted, that there is considerable debate in the literature with regards to these delta fit indices comparisons [cf. (82)], and that specific focus should also be placed on inspecting and giving substantial consideration to the parameter estimates of the various models and not just goodness-of-fit criteria when selecting the “final” model. The criteria chosen should be specified and justified by the researcher.

Morin et al. (20) further indicated that to retain an ESEM model for further analysis, several conditions need to be additionally met:

a. the ESEM model should ideally show better data-model fit than any other CFA model (however, if the factor correlations for the ESEM model are smaller than those of the CFA model, it should still be considered despite showing similar or worse fit). If not, the more parsimonious CFA model should be retained.
b. For normal (not bifactor) models, the ESEM model should show lower factor correlations than those produced by the CFA models.
c. The ESEM model should only show small to medium cross-loadings (<0.50). Should large cross-loadings exist, then there should be a theoretical explanation presented for such. These could potentially be explained by “wording” effects or some practical logic.
d. The estimated latent factors within the ESEM model should be well-defined (i.e., strong loadings, and loadings matching expectations).
e. Should there be multiple medium to large cross-loadings in the ESEM model, it could indicate support for the presence of a larger global factor, and therefore the bifactor ESEM model should be explored.
f. Additional factors to consider for bifactor ESEM models: This model could show a significantly better fit than any of the ESEM or CFA models because of the relatively large number of freed parameters. Therefore, there should be a well-defined G-Factor (where all items load significantly on such), and *reasonably* well-defined S-Factors (cross- and non-significant loadings are permitted). For bifactor models, model fit should not be the only indicator informing a decision to retain. Researchers should also closely inspect the parameter estimates before making final decisions.

*Ninth, for the best fitting model(s) the factor correlations should be computed and compared*. Morin [(20), p. 1060] argued that “in addition, the model comparison strategies typically advocated for contrasting alternative ESEM and CFA solutions highlight the critical role of the factor correlations, which directly indicate whether the cross-loadings have an impact on improving the factor definition.” Therefore, when choosing which model to retain, the factorial inter-correlations between latent factors for all the best fitting models (excluding the bifactor models) should be estimated and considered. This shows the level of unique distinction between factors. The model with the smallest factor correlation is usually retained, however, decisions should be based in the context of the other considerations (model fit, measurement quality, and parameter estimates) mentioned earlier. This step, however, cannot be done for bifactor models as the relationships between the specific and general factors are constrained to zero.

*Tenth, report and compare the item level parameters and levels of reliability for the best fitting measurement model*(s). This step aims to investigate the item level parameters and indicators of reliability for the best fitting models to further discriminate between the different models. This step is of particular importance when validating a psychometric instrument. However, item-level parameters should always be inspected, but may not be appropriate to report in studies unrelated to an instrument's validation. When validating an instrument, the standardized factor loadings, standard errors and item uniqueness should always be reported for the best fitting model when the paper's purpose is to validate an instrument. For the CFA models, the corrected item-total correlations (CITC) values represent each item's unique relationship with the overall factor on which it has been specified to load (83). Zijlmans et al. (83) argued that a CITC value bigger than 0.30 indicates that an item accurately represents the overall factor on which it specified. Note, that if a bifactor CFA model is retained, reviewers and editors may request additional information such as the Explained Common Variance (ECV), the H-factor, the Factor Determinacy indicator, the Item level ECV, the Percent of Uncontaminated Correlations (PUC), and the Average Relative Bias Parameters could also be reported as additional indicators of reliability and measurement quality [For a tutorial cf. (84)]. Further, the indicators for reliability should be decided and reported. To determine the level of reliability for the different factorial model, the following could be reported: point-estimate composite reliability [upper-bound; ρ > 0.80; (85)] or McDonald's Omega [ω > 0.70; (86)]. For the bifactor CFA models, the explained common variance (ECV) should be reported. A scale is regarded as essentially unidimensional when the general factor explains at least 70% of the total common variance. There are, however, no recommendations yet regarding how cross-loadings should be incorporated for bifactor ESEM models in Omega estimation [(20, 87)]. Morin et al. (20) suggest that these cross-loadings in bifactor ESEM models should, for the time being, be ignored when calculating Omega. It should also be noted, that for ESEM models, Omega cannot entirely reflect the reliability of a construct and should not be used as the only indicator. Rather, Omega should be used as an additional indicator to control for the fallible nature of psychological measurement and supplemented by other metrics of measurement quality.

#### Further or Additional Analysis

Should there be a need to conduct additional or more complex statistical estimations (e.g., latent growth modeling, invariance testing, multi-group analysis, structural path models, etc.), the ESEM-within-CFA approach should be employed. Here, the best fitting first-order ESEM model is respecified within a CFA structure, where all parameters of the ESEM model parameters are used as starting values for the ESEM-within-CFA model (39, 42). This would afford the opportunity to use the ESEM model as an input in a structural model.

## Tutorial and Results

### Conceptualization

**Figure A1:**
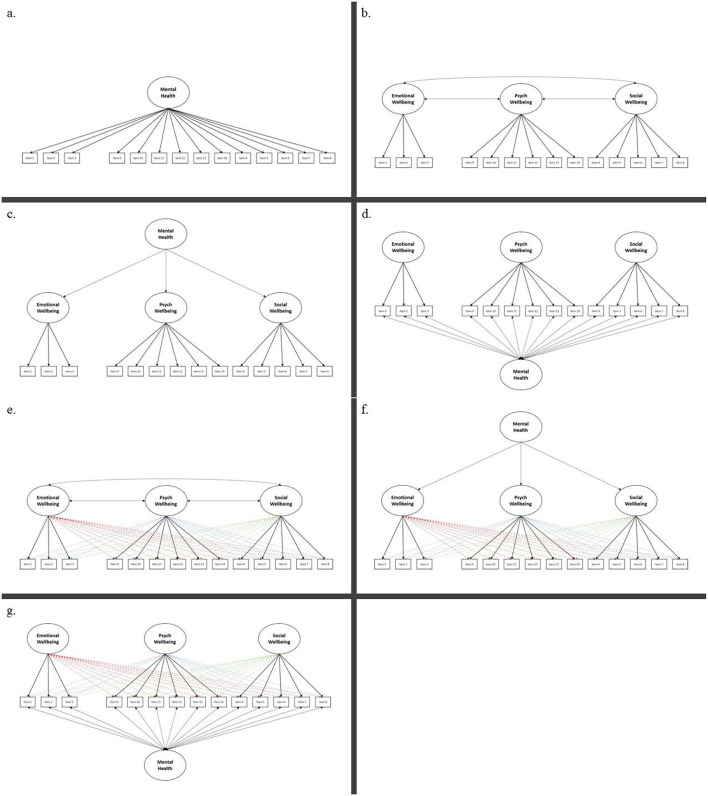
Graphical representations of the MHC-SF's various factorial models.

To determine the factorial validity of the MHC-SF, a competing measurement modeling strategy was employed comparing traditional CFA- with ESEM models. Based on the literature, the following models could be estimated:

0. Model 0: Unidimensional CFA Model of Overall Mental Health (58) (Figure A1a)
1. Model 1: Correlated Three First-Order CFA Model comprised of EWB, SWB, and PWB (14) (Figure A1b)
2. Model 2: Hierarchical CFA Model compromises a single Second-Order Factor of Mental Health, consisting of three first-order factors (47) (Figure A1c)
3. Model 3: Bifactor CFA Model of Overall Mental Health (15, 61) (Figure A1d)
4. Model 4: Correlated Three-factor First-Order ESEM Model comprised of EWB, SWB, and PWB (62) (Figure A1e)
5. Model 5: Hierarchical ESEM Model compromise of a single Second-Order Factor of Mental Health, made up of three first-order factors (47) (Figure A1f)
6. Model 6: Bifactor ESEM Model for Overall Mental Health (46, 61) (Figure A1g)
7. Model 7: Correlated Three-Factor First-Order ESEM within CFA Model^1^. Here, mental health is seen as the function of three independent first-order factors (as specified before). However, the starting values from Model 4 are used to constrain the items loadings for each independent factor.

### Sample Size Estimation

To estimate the minimum required sample size for the current study, the power and sample size approach of MacCallum et al. (71) was used for testing null hypotheses of not-good fit according to the RMSEA. Considering the previously proposed CFA models of the MHC-SF, ranging from a unidimensional to a bifactor CFA model (with the number of degrees of freedom ranging between 77 and 64), the minimum sample size would range between 249 and 278 to have 90% power to reject the hypothesis of not-close fit (RMSEA ≥ 0.05) at a 5% level of significance (88). Given that ESEM models have fewer degrees of freedom, slightly larger sample sizes are required for these models.

### Data Screening, Cleaning, and Preparation

The data was screened for potential issues (e.g., outliers, missing values, data quality) and prepared for further analysis. Based on the Mahalanobis' distance, two outliers were removed from the overall dataset [*p* <0.01; (76, 77)]. No missing values were present in the final dataset. Therefore, the final sample used for the study was *N* = 1,804.

### Determine the Most Appropriate Software, Estimator, Rotations, and Procedure

CFA and ESEM analyses were conducted using Mplus v 8.6 (50). To explore the factorial validity of the MHC-SF a competing measurement modeling strategy *via* structural equation modeling was used. The maximum likelihood (ML) estimation method was employed, given the relatively normal distribution of the data. For the ESEM models, an oblique target rotation was used and for the bifactor ESEM and bifactor CFA, a target orthogonal rotation was employed.

In order to determine the best-fitting model for the data, we estimated and sequentially compared several CFA (unidimensional, first-order factor, second-order factor, and bifactor) and ESEM (first-order ESEM, hierarchical ESEM, bifactor ESEM, and ESEM-within-CFA) models. The CFA models were specified according to the independent cluster modeling assumptions where items are only permitted to load onto their a priori theoretical factor, and cross-loadings were constrained to zero (79). For the bifactor model (B-CFA), we used a target orthogonal rotation. A general factor (G-factor) was specified, comprising all the items of the MHC-SF Further, three specific factors (S-factors) corresponding to the a priori theoretical dimensions of the MHC-SF were specified. For the ESEM models, we used a target rotation. Here, we specified items to load onto their a priori theoretical constructs where cross-loadings were freed but targeted to be as close to zero as possible (20). With the H-ESEM model, we explored a second-order factor structure where the original ESEM model was re-specified as a CFA model, in line with the ESEM-within-CFA framework. We used the non-standardized loadings from the ESEM model as starting values for the H-ESEM estimation. Factor variances were constrained to one, and one item per latent construct was constrained to be equal to the original ESEM item loading. Then we used the first-order factors to define a higher-order factor to determine the variance and the standardized path coefficient for each individual factor loading onto the overall Mental Health Factor. For the bifactor ESEM (B-ESEM) we followed a strategy similar to its B-CFA counterpart. However, in the B-ESEM, a target rotation was used where cross-loadings were freed and targeted to be as close to zero as possible. For all models, observed items were used as indicators for latent variables. For the ESEM-within-CFA model, mental health is seen as the function of three independent first-order factors, where the starting values from the initial ESEM model are used to constrain the items loadings for each independent factor. Here the original ESEM model is re-expressed within a CFA framework; as such, no rotation is necessary. Using De Beer and Van Zyl's (63) ESEM generator, we generated the Mplus syntaxes for the ESEM, B-ESEM and H-ESEM models.

### Determine Appropriate Goodness-of-Fit Indices and Indicators of Measurement Quality

To determine the best fitting measurement model, both goodness-of-fit indices and measurement quality indicators are used to discriminate between models. First, the Hu and Bentler (32) model fit criteria were used to establish data-model fit (cf. Table 2). Second, various indicators of measurement quality were used to further inspect and discriminate between models. Here, the standardized factor loadings (λ > 0.35), the item uniqueness (>0.10 but <0.90), levels of tolerance for cross-loadings, and overall *R*^2^ for each item was inspected. Only models that met both the measurement quality and goodness-of-fit criteria, were retained for further analyses.

### Estimate and Report the Model Fit Indicators for Competing CFA Models

Four CFA measurement models were estimated based on the different a priori factorial permutations of the MHC-SF found in the literature (see Point 1 of the Tutorial). In the model estimation, measured items were treated as continuous variables and used as indicators for the latent factors. No items were omitted, error terms were left uncorrelated and item parceling was not allowed. The following models were estimated in Mplus:

**Model 0: Unidimensional CFA Model of Overall Mental Health**. A unidimensional model for overall Mental Health (labeled “MENTAL”) was estimated, where all 14 items (MHC_1 to MHC_14) were specified to load directly on to such. This model acts as the baseline model for analyses.

**Model 1**: **Correlated Three First-Order CFA Model comprised of EWB, SWB and PWB**. A model was estimated where the MHC-SF is comprised of three first-order factors measured by 14 items: Emotional Well-being (labeled EMOTION comprised of items MHC_1 to MHC_3), Social Well-being (labeled SOCWELL comprised of items MHC_4 to MHC_8) and Psychological Well-being (labeled SOCWELL comprised of items MHC_9 to MHC_14). These factors were left to freely correlate.

**Model 2**: **A Hierarchical CFA Model compromised of a single Second-Order Factor of Mental Health, made up of three first-order factors**. Here, Mental Health was seen as a second-order factor that is a function of Emotional Well-being (Item: MHC_1 to MHC_3), Social Well-being (Items: MHC_4 to 8) and Psychological Well-being (Items: MHC_9 to MHC_14).

**Model 3: Bifactor CFA Model of Overall Mental Health**. MHC-SF was estimated to be comprised of a General Factor representing overall mental health (where all 14 items are specified to load onto such directly) which is distinct and independent from its three first-order factors Emotional Well-being (Item: MHC_1 to MHC_3), Social Well-being (Items: MHC_4 to 8), and Psychological Well-being (Items: MHC_9 to MHC_14). Here an Orthogonal Target rotation [ROTATION = TARGET (ORTHOGONAL)] was used and the relationships between specific and general factors were constrained to zero (this is automatically done in Mplus, but can also be manually specified by constraining the relationships between factors in the WITH statement to @0). The first factor loadings for each factor, which are automatically constrained to 1 by Mplus, were permitted to be freely estimated (indicated by the ^*^). The variances for each specific and general factor were constrained to 1 (indicated by @1). The initial results showed that the model couldn't converge, after which the iterations and starting values were increased. However, the results showed that item MHC_10 then produced a negative residual error variance. Kline (77), as well as Wong and Wong (81), indicated that in such cases, the residual error variance of the observed indicator should be constrained to be positive and slightly bigger than zero (MHC_10@0.03). This allowed the model to converge.

Table 3 provides a summary of the model fit indices for each of the estimated models. The results showed that none of the CFA models completely fitted the data based on the model fit criteria specified in Table 2. However, Model 3, the B-CFA model, partially met the goodness-of-fit criteria {$\chi_{\left(1,802\right)}^{2}$ = 868.74; *df* = 64; CFI = 0.94; TLI = 0.89; RMSEA = 0.08 [0.079, 0.088]; SRMR = 0.05}. The parameter estimates showed that this model produced a well-defined general factor (with all items λ > 0.35; small standard errors <0.04). Further, the specific factors were also relatively well-defined, with item loadings matching expectations for both the Emotional Well-being and Social Well-being subscales. However, items MHC_11, MHC_13, and MHC_14 on the Psychological Well-being Subscale showed non-significant specific factor loadings (*p* > 0.01). Further, items MHC_9, and MHC_12 only produced small, yet significant, factor loadings (λ <0.39) on the psychological well-being factor. Under normal circumstances, this model would therefore also be rejected for further consideration. However, for the purposes of this tutorial, Model 3 will be retained for further analyses to show how ESEM and CFA models can be compared.

**Table 3:** Competing CFA and ESEM measurement models.

**Model**	**Type**	**χ^2^**	***df***	**CFI**	**TLI**	**RMSEA**		**SRMR**	**AIC**	**BIC**	**aBIC**	**Meets Criteria**
***Confirmatory factor analytical models***												
Model 0	Unidimensional first-order factor model	2345.02	77	0.78	0.74	0.13	[0.128–0.132]	0.07	77662.47	77893.38	77759.95	No
Model 1	Three first-order factor model	1283.34	74	0.88	0.86	0.10	[0.091–0.100]	0.06	76606.79	76854.19	76711.23	No
Model 2	Second-order factor model	1283.34	74	0.88	0.86	0.10	[0.091–0.100]	0.06	76606.79	76854.19	76711.23	No
Model 3	Bifactor model	868.74	64	0.92	0.89	0.08	[0.079–0.088]	0.05	76212.19	76514.57	76339.84	No
***Exploratory structural equation models***												
Model 4	Three first-order ESEM	634.78	52	0.94	0.90	0.08	[0.073–0.084]	0.03	76002.23	76370.59	76157.73	Yes
Model 5	Higher-order ESEM	983.42	55	0.91	0.85	0.10	[0.091–0.102]	0.08	76344.87	76696.73	76493.40	No
Model 6	Bifactor ESEM	272.29	41	0.98	0.95	0.06	[0.050–0.062]	0.02	75661.74	76090.52	75842.76	Yes
Model 7	ESEM within CFA	634.78	52	0.94	0.90	0.08	[0.073–0.084]	0.03	76002.23	76370.59	76157.73	Yes

*χ^2^, Chi-square; df , degrees of freedom; TLI, Tucker-Lewis Index; CFI, Comparative Fit Index; RMSEA, Root Mean Square Error of Approximation [90%CI]; SRMR, Standardized Root Mean Square Residual; AIC, Akaike Information Criterion; BIC, Bayes Information Criterion; aBIC, Adjusted Bayes Information Criterion*.

### Estimate and Report the Model Fit Indicators for Competing ESEM Models

Next, a series of ESEM models were estimated based on the a priori CFA factorial structures of the MHC-SF found in the literature (see Point 1 of the Tutorial). Similar to the CFA models, measured items were tread as continuous variables and used as indicators for the latent factors. No items were omitted, and item parceling was not allowed. However, unlike the CFA models, cross-loadings were permitted but targeted to be close to zero. The De Beer and Van Zyl [(63); http://www.surveyhost.co.za/esem/] ESEM syntax generator was used to create the syntaxes needed to run the ESEM, B-ESEM, H-ESEM and ESEM-within-CFA models. For the purposes of this tutorial, we will walk readers through each step of the estimation process, from generating the codes *via* the tool to how it should be presented and interpreted. First, a general overview and step by step guide on using the tool will be presented. Second, the tool will be applied to the MHC-SF dataset to generate the results.

### Overview and Purpose of the De Beer and Van Zyl (63) ESEM Code Generator

The purpose of the De Beer and Van Zyl (63) ESEM tool is to aid researchers to generate the Mplus syntaxes needed to run several complex ESEM models. Estimating ESEM models within Mplus is rather complex and could become rather tedious. The tool was developed to intuitively guide researchers to generate the Mplus syntaxes needed to estimate normal ESEM-, bifactor ESEM-, Hierarchical ESEM (H-ESEM), and ESEM-within-CFA models. This tool is based on the ESEM estimation procedure discussed in Asparouhov and Muthén (30) and demonstrated by Howard et al. (45) for bifactor ESEM and Morin and Asparouhov (44) for H-ESEM and ESEM-within-CFA models.

Estimating these ESEM models can be done in four relatively easy steps:

1. **STEP 0: Navigate any web browser to** http://www.surveyhost.co.za/esem/
2. **STEP 1: Specify a CFA Model**. Provide the Mplus syntax code for a traditional First-Order CFA Factorial model and Click Continue to generate the syntaxes.
3. **STEP 2: Generate, Copy, and Run the ESEM Syntaxes in Mplus**. Copy the syntax generated for the Regular ESEM or bifactor ESEM solution into Mplus and run these models.
4. **STEP 3 (Optional): Generate, Copy, and Run the Syntax for H-ESEM/ESEM-within-CFA Models**. Upload the Mplus Output produced in STEP 2 to generate the syntaxes for the H-ESEM- and ESEM-within-CFA models and click continue.

#### Step 1: Specify a CFA Model

**Figure 1:**
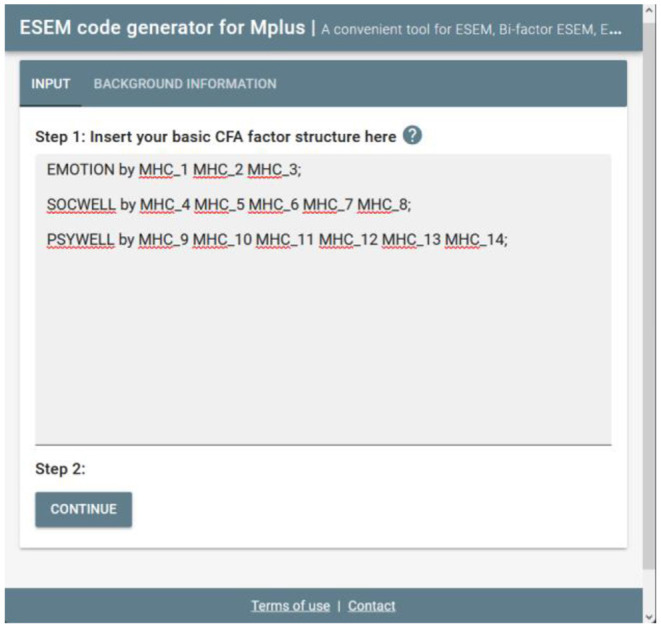
Step 1: specify a CFA model.

Once users have directed their browser to the online tool, they will be requested to specify a basic CFA factorial model. This tool only accepts the syntax commands for Mplus v.6 and above. Specify a basic first-order CFA factorial (measurement) model in the Mplus language on the INPUT command (cf. Figure 1). This can be done by using the BY command in Mplus (e.g., EMOTION by Item1 Item2 Item3;). Ensure that all first-order factors are correctly specified, and the command closes with a ‘;'. Once done, the researcher should click continue to generate the Mplus syntaxes.

#### Step 2: Generate-, Copy-, and Run the ESEM Syntaxes in Mplus

**Figure 2:**
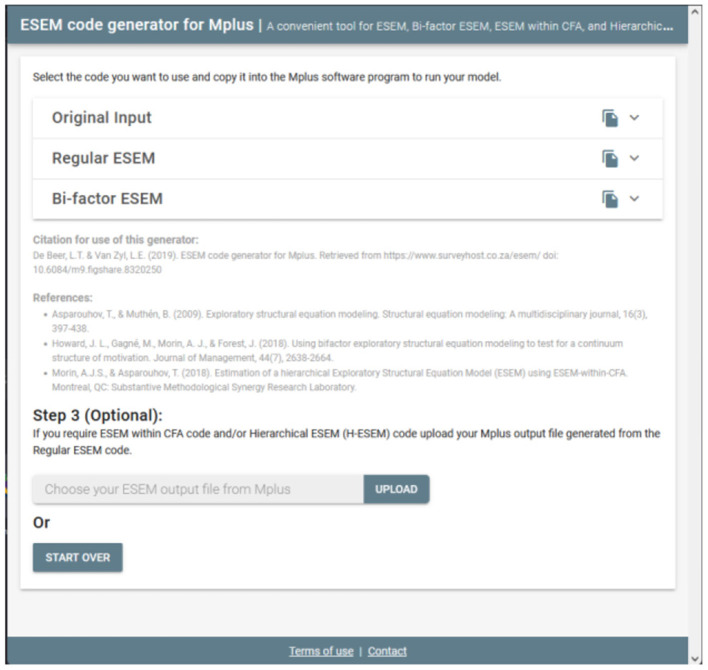
Step 2.1: Generated ESEM and bifactor ESEM Syntaxes.

**Figure 3:**
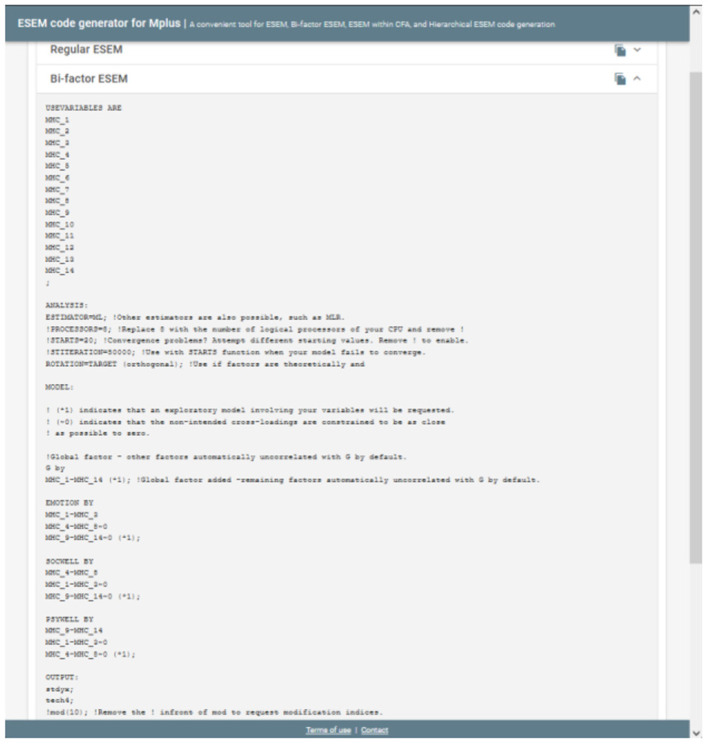
Step 2.2: Example output for the bifactor ESEM Syntax.

Upon clicking continue, the user will be redirected to a new page whereby the Original Input is provided as well as the regular- and bifactor ESEM syntaxes generated (see Figure 2). The syntax provides the correctly specified ESEM models and brief descriptions of each command for ease of reference (see Figure 3 as an example). The appropriate syntax required by the researcher should then be copied and pasted in Mplus. Researchers should, however, still specify (1) the name- or location of the dataset next to the FILE IS command, (2) populate the variables names from the dataset under the NAMES ARE command, (3) specify how missing values are labeled under the MISSING ARE ALL (-XX) command, (4) make choices in the ANALYSIS command relating to the estimation method, and rotation type, and (5) any additional outputs required [cf. (50) for an outline of different output commands]. Once these factors have been clarified, the researcher can run both models in Mplus. The output of the ESEM model should be saved, as this will be used as input for the next step, should users want to generate H-ESEM or ESEM-within-CFA models.

#### Step 3 (Optional): Generate, Copy, and Run the Syntax for H-ESEM or ESEM-Within-CFA Models

**Figure 4:**
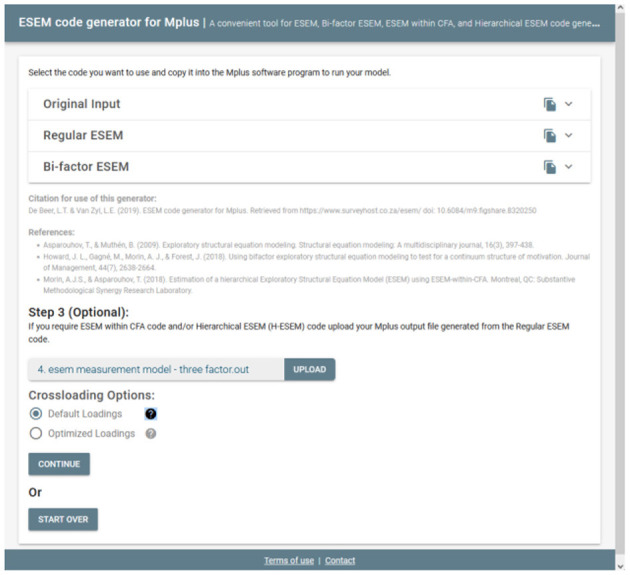
Uploading ESEM output and specifying cross-loadings option.

Researchers may also be interested in estimating more complex ESEM models such as H-ESEM or ESEM-within-CFA models. These models require that the starting values for both be changed from the defaults. The non-standardized factor loadings from the original ESEM model should be used as starting values for the H-ESEM and ESEM-within-CFA model estimation syntaxes. Factor variances are also constrained to one, and one item per latent construct is constrained to be equal to the original ESEM item loading. Then we used the first-order factors to define a higher-order factor to determine the variance and the standardized path coefficient for each individual factor loading onto a higher-order factor. For the ESEM-within-CFA model, the regular ESEM model is re-expressed as a CFA model. This model employs the unstandardized factor- and cross-loadings estimated from the regular ESEM model as starting values (denoted by the ^*^ command). First-order factor variances are again freely estimated, whereas the higher-order factor is constrained to 1 in order to identify the model. Furter, in this model, one item per first-order factor has all its factor loadings constrained to equal that of the original ESEM values denoted by the @ command. Researchers can do this manually, however, this leaves room for error. The tool aids researchers to generate these syntaxes, by requesting that the original Mplus output from the ESEM model generated in Step 3 be uploaded. Researchers should click on the UPLOAD button and direct their explorer to the output file from the original ESEM model (see Figure 4)^2^.

**Figure 5:**
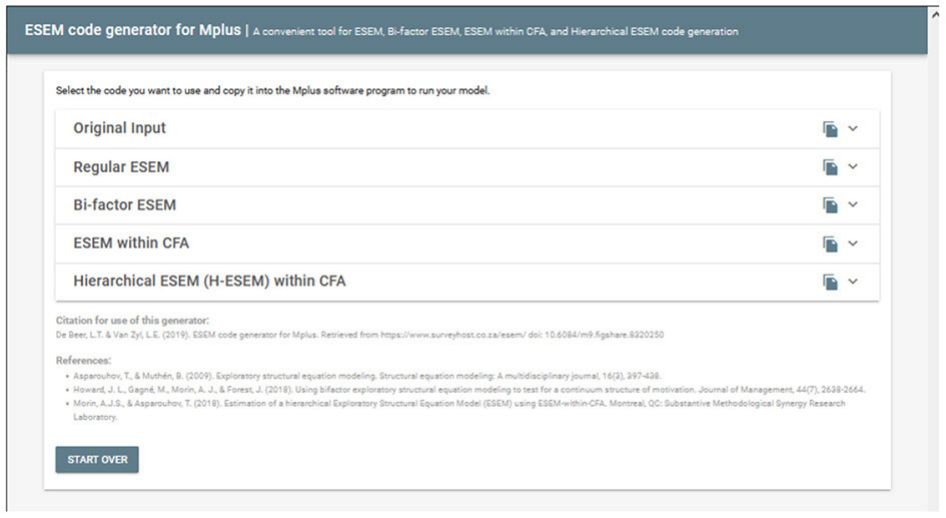
Step 3: H-ESEM or ESEM-within-CFA models codes generated.

Once selected, users can select from two cross-loading options: (a) the *Default Loadings* which uses the largest factor loading for each factor and fixes it as the cross-loading for each factor or (b) *Optimized Loadings* whereby the script attempts to find the most optimal loadings from the specific original factor where it also has the smallest cross-loadings to fix in the model. The latter is still an experimental feature and novices are encouraged to use the *Default Loadings* option. Once the cross-loadings option is selected, researchers can click on CONTINUE to generate the new syntaxes (kindly note that no information about the data is captured or stored on the server). This will then redirect the user to a new page where two additional ESEM syntax options have been generated (see Figure 5). These syntaxes can then be copied and pasted into Mplus to be run. The tool will be practically applied, and example codes are shown in the next section.

### Estimate and Report the Results for Competing ESEM Models

After the four CFA models were estimated, four ESEM models were tested against the data. These ESEM models follow the same theoretical structure of the CFA models, however, cross-loadings were permitted but constrained to be as close to zero as possible. The syntaxes for these ESEM models were generated with the ESEM Code Generator (63). The following ESEM models were estimated in Mplus:

#### Model 4: Correlated Three-Factor First-Order ESEM Model Comprised of Emotional-, Social-, and Psychological Well-Being

**Figure 6:**
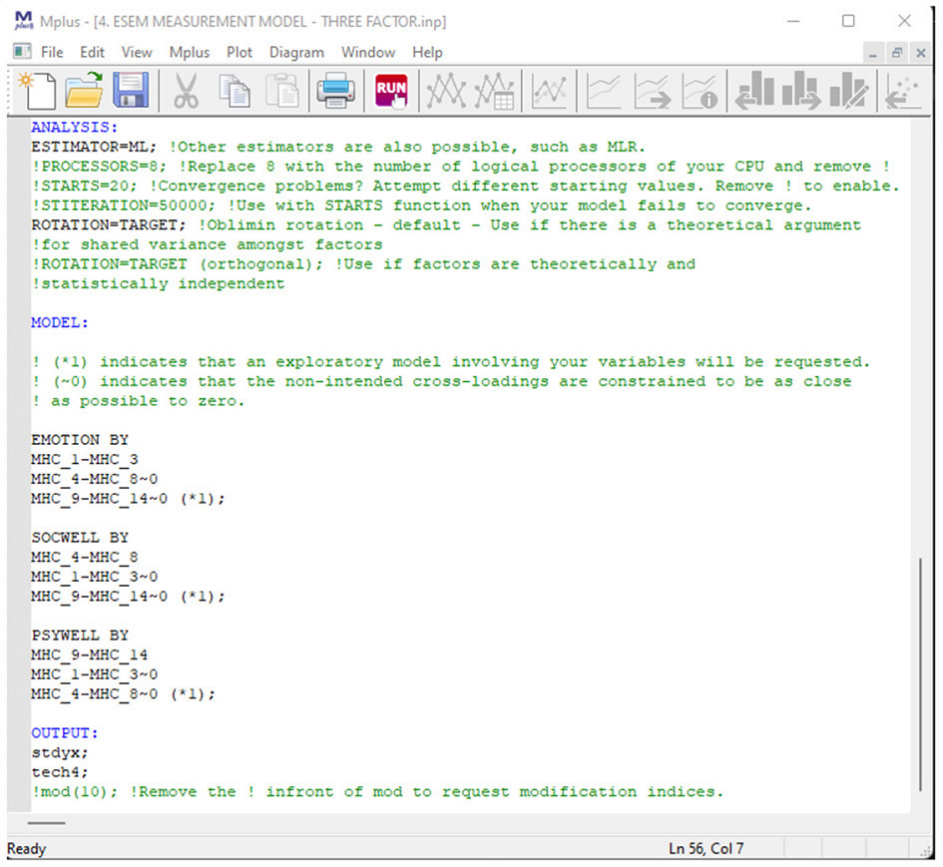
Mplus syntax for a three first-order ESEM model of mental health.

This model assumes that Emotional- (Targeted Items: MHC_1 to MHC_3), Social- (Targeted Items: MHC_4 to 8), and Psychological well-being (Targeted Items: MHC_9 to MHC_14) are separate yet related components of mental health. In this model, items are targeted to load onto their a priori factorial model, but cross-loadings were permitted but targeted to be close to zero. The code generated to run the model in Mplus is presented in Figure 6.

#### Model 5: A Hierarchical ESEM Model Compromised of a Single Second-Order Factor of Mental Health, Made Up of Three First-Order Factors

**Figure 7:**
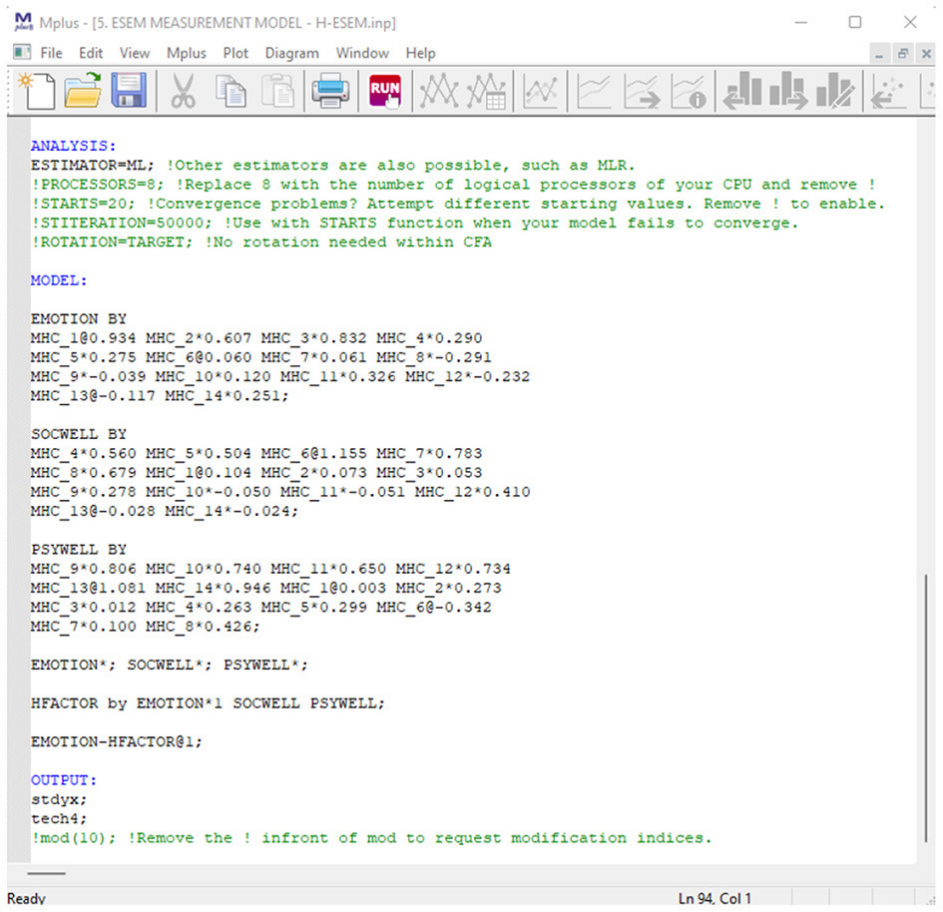
Mplus syntax for a H-ESEM model of mental health.

Mental health was specified as a second-order ESEM model that is a function of EWB (Item: MHC_1 to MHC_3), SWB (Items: MHC_4 to 8), and PWB (Items: MHC_9 to MHC_14). Again, items were specified to load directly onto their a priori first-order factors. Cross loadings were again permitted but constrained to be as close to zero as possible. The ESEM-within-CFA estimation procedure was used to construct the higher-order factorial model. Here the starting values for each item was constrained to be the same as the unstandardized factor- and cross-loadings estimated from the Regular ESEM Model (Model 4). The first-order factor variances were freely estimated and that of the higher-order Mental Health Factor was constrained to 1. Further, for the first-order factor, one item per factor is constrained to produce exactly the same loadings and cross-loadings as the ESEM Model 4. Finally, the Higher-Order Factor Mental health (or HFACTOR in the syntax in Figure 7) was specified as being comprised of freely estimated first-order factors.

#### Model 6: Bifactor ESEM Model for Overall Mental Health

**Figure 8:**
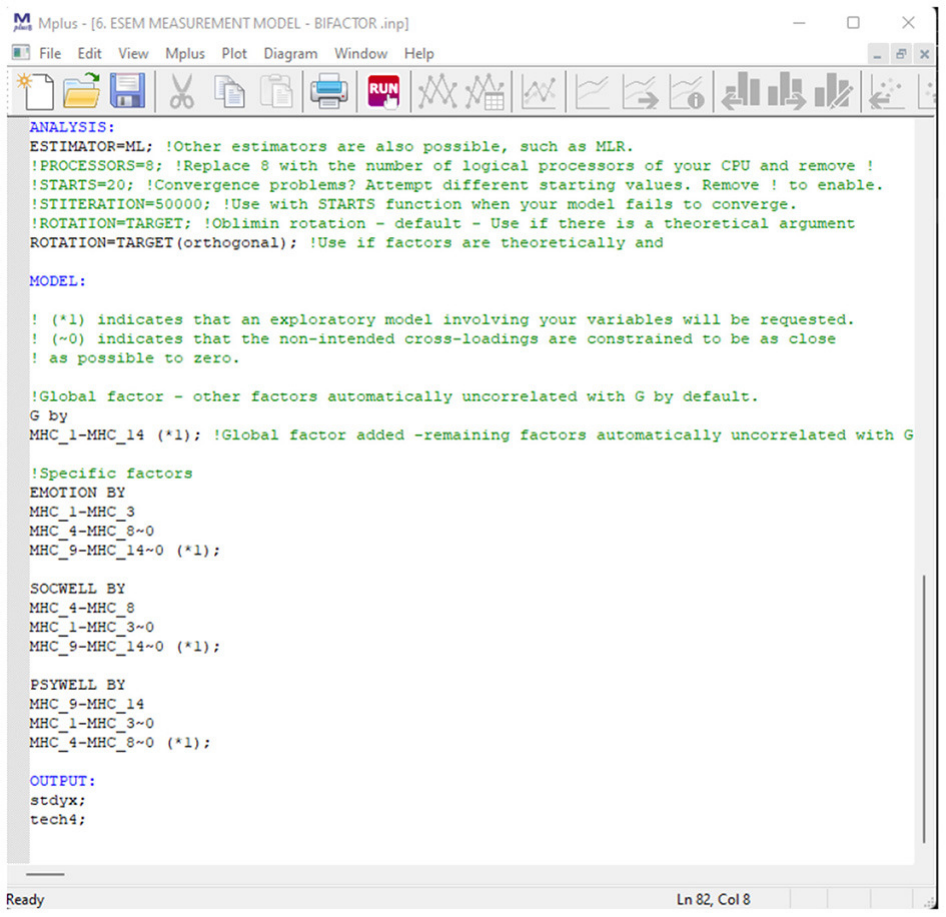
Mplus syntax for a bifactor ESEM model of mental health.

Similar to Model 3, the MHC-SF was specified to be comprised of a General Factor representing overall mental health (where all 14 items are specified to directly load onto such) which is distinct and independent from its three first-order factors emotional- (Target Item: MHC_1 to MHC_3), social- (Target Items: MHC_4 to 8), and psychological well-being (Target Items: MHC_9 to MHC_14). Target items were specified to load directly on their a priori factorial models but cross-loadings on the specific factors were permitted but constrained to be as close to zero as possible. Figure 8 provides a screenshot of the Mplus Syntax.

#### Model 7. A Correlated Three-Factor ESEM-Within-CFA Model

**Figure 9:**
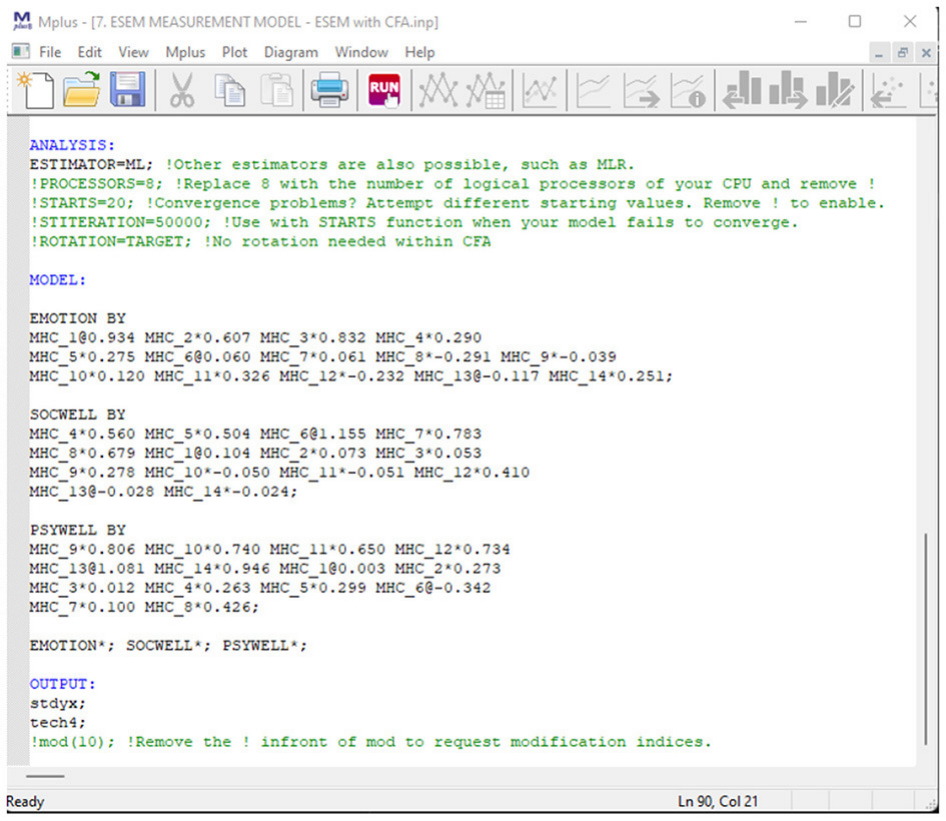
Mplus syntax for the ESEM-within-CFA model of mental health.

Here, mental health is seen as the function of three independent first-order factors (as specified before). Within this model, the Regular ESEM Model (Model 4) is re-expressed as a CFA model, where the starting values from Model 5 are used to constrain the items loadings for each independent factor. The variances of the three first-order factors (emotional-, social-, and psychological well-being) are freely estimated. This model is not a separate or different type of ESEM model that should be contrasted/compared. This model is only specified to be used for more complex follow-up analysis. It's only estimated and compared here to demonstrate the tool. The Syntax is presented in Figure 9.

The model fit indices of the ESEM models are also captured and summarized in Table 4. Unlike the CFA models, the results showed that *Model 4* [$\chi_{\left(1,802\right)}^{2}$ = 634.78; *df* = 52; CFI = 0.94; TLI = 0.90; RMSEA = 0.08 [0.073, 0.084]; SRMR = 0.03; AIC = 76002.24; BIC = 76370.59], *Model 6* [$\chi_{\left(1,802\right)}^{2}$ = 272.285; *df* = 41; CFI = 0.98; TLI = 0.95; RMSEA = 0.06 [0.050, 0.062]; SRMR = 0.02; AIC = 75661.74; BIC = 76090.56], and *Model 7* [$\chi_{\left(1,802\right)}^{2}$ = 634.78; *df* = 52; CFI = 0.94; TLI = 0.90; RMSEA = 0.08 [0.073, 0.084]; SRMR = 0.03; AIC = 76002.24; BIC = 76370.59] all fitted the data. Further Model 4 (the original ESEM model) and Model 7 (the ESEM-within-CFA models) produced, as intended, the same results. Further, by inspecting the parameter estimates, the results showed that all models, except Model 5, showed the expected results with target items loading significantly on their respective factors (λ > 0.35; small standard errors <0.04). Given that Model 5 did not meet either the model fit or measurement quality criteria, it was disregarded for further analysis.

**Table 4:** Comparing CFA and ESEM models.

	**Model**	**Model comparison**	**Δχ^2^**	**Δ*df***	**ΔCFI**	**ΔTLI**	**ΔRMSEA**	**ΔSRMR**	**ΔAIC**	**ΔBIC**	**Differences**
Model 3	Bifactor model	M6 vs. M3	−596.46	27	0.056	0.061	−0.027	−0.028	−550.46	−424.01	Yes
		M4/7 vs. M3	−233.96	−12	0.022	0.012	−0.004	−0.016	−209.96	−143.99	Yes
Model 4	Three first-order ESEM	M6 vs. M4	−362.50	39	0.034	0.049	−0.023	−0.012	−340.50	−280.02	Yes
Model 6	Bifactor ESEM	M6 vs. M7	−362.50	390	0.034	0.049	−0.023	−0.012	−340.50	−280.02	Yes
Model 7	ESEM within CFA	M7 vs. M4	0.000	0	0.000	0.000	0.000	0.000	0.00	0.00	No

*χ^2^, Chi-square; df , degrees of freedom; TLI, Tucker-Lewis Index; CFI, Comparative Fit Index; RMSEA, Root Mean Square Error of Approximation [90%CI]; SRMR, Standardized Root Mean Square Residual; AIC, Akaike Information Criterion; BIC, Bayes Information Criterion*.

### Compare CFA and ESEM Models to Determine the Best-Fitting Model for the Data

The next step is to contrast and compare the retained CFA and ESEM models. From the previous sections the bifactor CFA Model (Model 3) as well as the Three-First Order Factor ESEM (Model 4), bifactor ESEM (Model 6)„ and ESEM-within-CFA (Model 7) were retained. These competing Models are further compared based on their Model Fit Indices, and the results summarized in Table 4. The results showed that *Model 6* fitted the data significantly better than *Model 3* (Δχ^2^ = −596.46; Δ*df* = 27; ΔCFI = 0.06; ΔTLI = 0.06; ΔRMSEA = −0.03; ΔSRMR = −0.03; ΔAIC: −550.45; ΔBIC: −424.00), and Model 4 and Model 7 (Δχ^2^ = −362.498; Δ*df* = 39; ΔCFI = 0.03; ΔTLI = 0.05; ΔRMSEA = −0.02; ΔSRMR = −0.01; ΔAIC: −340.50; ΔBIC: −280.23). The ESEM Model 6 is therefore retained for further comparisons and the CFA Model 3 just retained for purposes of the tutorial.

### Factor Correlations

The factor correlations between factors for the best fitting measurement models should be estimated and compared in the next step. Models where the lowest correlations between factors are shown, show that these models are able to better discriminate between factors. The model with the lowest factor correlations should be retained (20). However, given that a bifactor ESEM and bifactor CFA model fit the data the best in the current sample, inter-factor correlations cannot be computed as relationships are constrained to zero.

### Item Level Parameters, Standardized Factor Loadings, and Reliability

In the final step, item level parameters and reliability indicators are reported. For the sake of transparency in this tutorial, item-level descriptive statistics (means, standard deviations, skewness, kurtosis) are also reported. Further, indicators of measurement quality (standardized factor loadings, standard errors, item-level residual variances, and item level *R*^2^) and levels of reliability (omega and CITC) were computed for both the bifactor CFA (Model 3) and bifactor ESEM Models (Model 6).

The results summarized in Table 5 show that items were relatively normally distributed [Skewness and Kurtosis < +2; −2: (89)], that each item was adequately associated with its overall a priori factor [CITC *r* > 0.30: (83)] and that the general and specific factors for both the ESEM and CFA models showed to be reliable.

**Table 5:** Item level descriptive statistics, factor loadings, and reliability indicators of Model 3 and Model 6.

**Factor**	**Item**	**Mean**	**SD**	**Skewness**	**Kurtosis**	**CITC**	**Model 3–bifactor CFA model**					**Model 6–bifactor ESEM model**										
							**G**_**factor**_		**S**_**factor**_			**G**_**factor**_				**EWB S_**factor**_**		**SWB S**_**factor**_			**PWB S_**factor**_**	
							**λ**	**S.E**.	**λ**	**S.E**.	**δ**	**λ**	**S.E**.	**R^**2**^**	**λ**	**S.E**.	**λ**	**S.E**.	**R^**2**^**	**λ**	**S.E**.	**δ**
***Emotional well-being***																						
	MHC_1	4.41	1.17	−0.78	0.26	0.72	**0.57**	0.02	**0.59**	0.02	0.32	**0.57**	0.02	0.33	**0.62**	0.02	−0.01	0.02	0.00	−0.05	0.02	0.29
	MHC_2	4.85	1.11	−1.11	1.11	0.65	**0.63**	0.02	**0.40**	0.02	0.44	**0.62**	0.02	0.39	**0.41**	0.02	0.02	0.02	0.00	0.08	0.02	0.44
	MHC_3	4.72	1.04	−0.92	0.90	0.72	**0.55**	0.02	**0.64**	0.02	0.29	**0.54**	0.02	0.29	**0.62**	0.02	−0.01	0.02	0.00	0.10	0.02	0.31
***Social well-being***																						
	MHC_4	3.60	1.46	−0.20	−0.91	0.54	**0.59**	0.02	**0.23**	0.03	0.60	**0.58**	0.02	0.33	0.12	0.02	**0.24**	0.03	0.06	−0.11	0.02	0.58
	MHC_5	3.75	1.62	−0.33	−1.07	0.47	**0.53**	0.02	**0.18**	0.03	0.69	**0.52**	0.02	0.27	0.11	0.02	**0.18**	0.03	0.03	−0.16	0.02	0.67
	MHC_6	2.35	1.29	0.60	−0.61	0.54	**0.38**	0.02	**0.65**	0.04	0.44	**0.38**	0.02	0.15	0.02	0.02	**0.62**	0.03	0.38	−0.10	0.02	0.46
	MHC_7	3.54	1.36	−0.21	−0.83	0.52	**0.48**	0.02	**0.45**	0.03	0.57	**0.47**	0.02	0.22	0.02	0.02	**0.46**	0.03	0.22	0.10	0.02	0.55
	MHC_8	3.40	1.46	−0.13	−1.01	0.46	**0.45**	0.02	**0.36**	0.03	0.67	**0.46**	0.02	0.21	−0.18	0.02	**0.40**	0.03	0.16	0.13	0.03	0.58
***Psychological well-being***																						
	MHC_9	3.99	1.35	−0.59	−0.43	0.64	**0.69**	0.02	**−0.17**	0.02	0.50	**0.71**	0.02	0.50	−0.07	0.02	0.16	0.02	0.03	**0.23**	0.04	0.42
	MHC_10	4.71	1.16	−1.09	1.02	0.58	**0.65**	0.02	**−0.74**	0.02	0.02	**0.67**	0.03	0.45	0.02	0.02	−0.04	0.02	0.00	**0.47**	0.05	0.33
	MHC_11	4.61	1.23	−0.84	0.16	0.60	**0.67**	0.02	−0.02	0.02	0.55	**0.67**	0.02	0.44	0.17	0.02	−0.08	0.02	0.01	−0.01	0.04	0.52
	MHC_12	3.36	1.58	−0.07	−1.14	0.49	**0.58**	0.02	**0.21**	0.02	0.62	**0.59**	0.02	0.35	−0.19	0.02	0.12	0.02	0.01	**−0.34**	0.05	0.48
	MHC_13	4.08	1.35	−0.53	−0.45	0.67	**0.71**	0.01	0.01	0.02	0.50	**0.73**	0.01	0.54	−0.12	0.02	−0.07	0.02	0.00	−0.06	0.05	0.44
	MHC_14	4.37	1.44	−0.80	−0.27	0.67	**0.76**	0.01	−0.02	0.02	0.42	**0.76**	0.01	0.57	0.09	0.02	−0.08	0.02	0.01	−0.02	0.04	0.42

***Bold items**, Significant target loadings (*p* <0.05); Underlined items indicate cross-loading items; S.E., standard error; CICT, Corrected item total correlation; λ, Standardized factor loadings; S.E., Standard Error; δ, Item Uniqueness.*

Further, both Model 3 and Model 6 produced well-defined general factors (with all items λ > 0.35; small standard errors <0.04). Further, the specific factors were also relatively well-defined with item loadings matching expectations for both the Emotional Well-being and Social Well-being subscales. However, items MHC_11, MHC_13, and MHC_14 on the Psychological Well-being Subscale for both models showed non-significant factor loadings (*p* > 0.01). Further, items MHC_9, and MHC_12 only produced small, yet significant, factor loadings (λ <0.39) on the Psychological Well-being factor for both models.

Finally, the level of reliability of the two models and their subscales were computed using Dueber's (84) calculator. The results, summarized in Table 6, show that the proportion of the common variance explained by the specific and general factors (ECV), and the overall omega produced similar results. The bifactor ESEM model did, however, produce slightly higher ECV values for the General Mental Health Factor (ECV = 0.70), and the Emotional- (ECV = 0.48) and Social well-being (ECV = 0.42) subscales. Both models produce similar, if not equivalent, levels of reliability with Omega exceeding the suggested cut-off criteria. However, when accounting for the presence of the general factor, the specific factors for neither models produced adequate Omega_hs_ levels (Omega_hs_ <0.70).

**Table 6:** Reliability estimates and explained common variance.

	**Model 3: bifactor CFA**			**Model 6: bifactor ESEM**		
	**ECV**	**Omega**	**Omega hs**	**ECV**	**Omega**	**Omega hs**
General factor	0.68	0.92	–	0.70	0.92	–
Emotional well-being	0.47	0.85	0.39	0.48	0.84	0.40
Social well-being	0.41	0.76	0.28	0.42	0.76	0.29
Psychological well-being	0.19	0.87	0.03	0.12	0.86	0.01

Taken together, the results show that the bifactor ESEM model fitted the data proportionally better than the bifactor CFA model and produced slightly better parameter estimates. As such, the bifactor ESEM model is retained for potential further analyses.

### Further Analysis: Demonstration of ESEM-Within-CFA in a Structural Model

Although the results showed that the bifactor ESEM model should be retained for further analysis, it does not afford the possibility to demonstrate the full usefulness or function of the ESEM-within-CFA framework [cf. (24)]. As stated previously, regular ESEM models can not directly be used within more complex estimation procedures (e.g., structural models) as they would produce convergence problems, and therefore the ESEM-within-CFA framework should be used to re-specify an ESEM model within a CFA framework. Within the current tutorial, the ESEM-within-CFA approach was already used to estimate the higher-order ESEM model (Model 5), however, the three first-order factorial ESEM model (Model 4) fitted the data better. Therefore, to demonstrate ESEM-within-CFA function, Model 3 was respecified as an ESEM-within-CFA model to produce Model 7. This model, which should produce exactly the same model fit statistics as the normal ESEM model, could therefore be used for more complex analysis.

**Figure 10:**
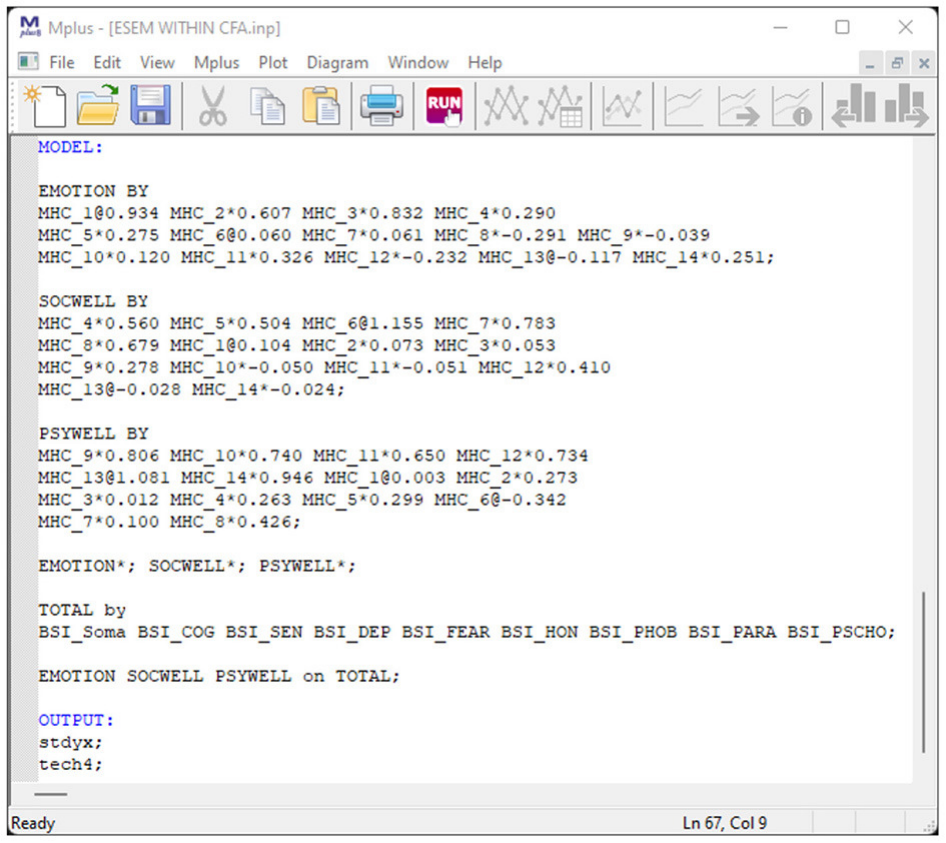
Mplus syntax for the relationship between BSI and ESEM-within-CFA model of mental health.

Based on Westerhof and Keyes' (54) assertion that mental health and mental illness are on separate, yet related, continuums, the relationship between common mental health problems, and mental health was investigated. As such, a structural path model was estimated based on the three first-order ESEM-within-CFA model of mental health and a traditional CFA model for mental illness as measured by the Brief Symptoms Inventory (cf. Figure 10). In this model, the common mental health problems were specified as the exogenous (input) factor and regressed on the three components of mental health (emotional well-being, social well-being, and psychological well-being) as the endogenous (outcome) factors.

The structural model showed acceptable fit: χ^2^_(202, N = 1, 084)_ = 1,419.42 (*p* < 0.01; TLI = 0.92; CFI = 0.93; RMSEA = 0.06 [0.055–0.061]; SRMR 0.03; AIC = 116579.93; BIC = 117113.22; aBIC = 116805.05). The results showed that overall common mental health problems explained 35.5% of the variance in Emotional Well-being (β: −0.60; S.E: 0.02; *p* < 0.01), 1.5% in Social Well-being (β: −0.12; S.E: 0.03; *p* < 0.01) and 8.9% in Psychological Well-being (β: 0.30; S.E: 0.03; *p* < 0.01). This implies that higher levels of overall common mental health problems is associated with lower levels of emotional-, psychological-, and social well-being.

## Conclusion

The purpose of this paper was to illustrate the applicability of ESEM as an alternative to traditional CFA approaches when evaluating the factorial validity of an instrument. By using the MHC-SF as an example, we provided (a) a brief overview of ESEM (and different ESEM models), (b) structured guidelines on how to estimate, compare, report, and interpret ESEM models, and (c) a step-by-step guide on how to produce ESEM syntaxes for Mplus with an innovative online tool. The results of this study highlight the value of ESEM, over and above that of traditional confirmatory factor analytical approaches. The study results also show practical implications for measuring mental health with the MHC-SF, by illustrating that a bifactor ESEM model fits the data significantly better than any other empirical model.

This tutorial demonstrates that restrictive CFA models for the MHC-SF, where items are constrained to only load onto their respective subscales, are insufficient to provide good model fit and adequately describe the data. Specifically, the results showed that neither the original correlated three-factor CFA model for mental health (55), nor a higher-order factorial model (58) could be confirmed. Similarly, the bifactor CFA model proposed by Jovanovic (15), where mental health is seen as a general factor, and emotional-, social-, and psychological well-being are positioned as specific factors, only partially fitted the data. Without specific- and deliberate modifications (e.g., correlating error variances of several items), the data would not adequately represent these models. Therefore, when cross-loadings are constrained, it undermines the measurement model.

In contrast, except for the Hierarchical ESEM Model, all ESEM models seemed to fit the data. Comparatively, the three first-order ESEM models comprised emotional-, psychological-, and social well-being. The bifactor ESEM model fitted the data significantly better than the CFA models. Therefore, it would seem that the less restrictive models, that accounts for small cross-loadings between items, overcame the limitations to the overly restrictive CFA models in terms of both model fit and indicators of measurement quality. These findings are in line with other ESEM studies on the MHC-SF that reported generally better data-model fit and stronger factor loadings compared to CFA models (46, 61, 62). Further, our results are in line with those of Lamborn et al. (61) that showed significant support for a bifactor ESEM model. Here, mental health is better represented by an overall general mental health factor, which is different from, three specific factors of well-being. The general mental health factor accounted for a large proportion of the variance and showed adequate levels of reliability. The three specific factors were also adequately represented by the data, however, these only accounted for a small proportion of the overall variance. This is similar to the findings of Lamborn et al. [(61), p. 15], who also argued that researchers should, therefore, “exercise caution when using and interpreting mean specific subscores” of these bifactor models. The relative strength of the g-factor in this model is not surprising, as studies comparing hedonic and eudaimonic conceptualisations of mental health have shown consistent support for a more general conceptualization of well-being and mental health (90). Our results, therefore, support ever-growing evidence in the literature for a tripartite model of mental health (61).

Therefore, mental health researchers are encouraged to incorporate ESEM into their measurement modeling strategies and structurally compare such to traditional CFA approaches. Given that mental health (and its three components) are structurally and theoretically linked, it requires researchers to apply less restrictive ESEM models, because cross-loadings between factors are inherently expected (62). Failure to employ these measures may lead to “a premature dismissal of central aspects of mental health” [(62), p. 11] and create unwarranted speculations within the literature.

Although this tutorial illustrated the applicability of ESEM when attempting to explore the factorial validity of an instrument, it is not without its limitations. The data employed in this study is derived from self-report measures, is cross-sectional, and participants were remunerated for their responses. This implies that there may be some biased results, and responses may not accurately represent reality. Further, there is currently no accepted means to account for the cross-loadings in ESEM models when estimating the scales' reliability. Therefore, omegas produced may not adequately represent the reliability of ESEM models, and direct comparisons of these with traditional CFA models should be made with caution. Further, the cut-off criteria to establish model fit are still based on CFA principles and the maximum likelihood estimation method. Although researchers are cautioned against stringent reliance on rules of thumb and cut-off scores, they do provide some form of standardization that aids in interpreting results. Therefore, simulation studies are required to determine the relevance and functioning of these goodness-of-fit criteria in relation to ESEM model estimation. Further, given the flexibility of ESEM, it is difficult to demonstrate its full potential and to articulate a full use case with a single dataset clearly. This tutorial provides a gentle introduction to the estimation and exploration of the factorial structure of a single instrument. Future tutorials should aim to incorporate more complex use cases such as auto-regressive modeling, measurement invariance, cross-lagged panel analysis, and the like.

Notwithstanding these limitations, our tutorial provides an illustrative example of approaching and estimating ESEM models with Mplus through an easy to use code generating tool. It also attempted to provide some suggested guidelines for approaching an ESEM related study. We hope that this tutorial and tool will aid researchers in incorporating ESEM into their model estimation approaches and provide more realistic and thorough evidence of their findings.

## Data Availability Statement

The original contributions presented in the study are included in the article/Supplementary Material, further inquiries can be directed to the corresponding author.

## Ethics Statement

Ethical review and approval was not required for the study on human participants in accordance with the local legislation and institutional requirements. Written informed consent to participate in this study was provided by the participants' legal guardian/next of kin.

## Author Contributions

LZ and PK conceptualized the tutorial. LZ analyzed the data, drafted the first version of the manuscript, and compiled the Supplementary Material. PK provided substantial conceptual input on the manuscript throughout the drafting process. All authors read through and approved the final manuscript before submission.

## Conflict of Interest

The authors declare that the research was conducted in the absence of any commercial or financial relationships that could be construed as a potential conflict of interest.

## Publisher's Note

All claims expressed in this article are solely those of the authors and do not necessarily represent those of their affiliated organizations, or those of the publisher, the editors and the reviewers. Any product that may be evaluated in this article, or claim that may be made by its manufacturer, is not guaranteed or endorsed by the publisher.

## Footnotes

^1^Note that the ESEM-with-CFA is not an alternative factorial model. It's specified as a means to conduct more complex analysis as discussed prior. This model is only specified and compared to demonstrate the full capability of the De Beer and Van Zyl (63) ESEM code generator and to demonstrate that no differences exist between it and Model 4.

^2^It is, however, important to note that in the ESEM within CFA code generator that all referent indicators are fixed (@) in all the factors to the starting values to aid in convergence. Therefore, factor variances are freed (as indicated by the ^*^; e.g., EMOTION^*^). If these factor variances are manually constrained to 1 (e.g., EMOTION@1;) then the starting value of the referent indicator in its original factor should then be freed (^*^). Either specification should result in the same degrees of freedom, model fit and parameter estimates.

## Appendix

**Table A1:** Summary of ESEM estimation guidelines.

**Step**	**Description**	**What to consider or report**	**Reported in manuscript**	**Where to report**
***Planning Phase***				
1	Develop a clear theoretical model for the instrument based on the available literature	Briefly discuss alternative, theory-informed (CFA) factorial models which the instrument could manifest as. If factors within a CFA model are conceptually related, then there is also an argument for an ESEM model with a similar structure. Should a global factor be expected, a bifactor CFA should be described and an associated bifactor ESEM model mentioned.	Yes	Literature review
		For the validation of psychometric instruments, present clear hypotheses about the (CFA) factorial structure of the instrument and present alternative hypotheses that the ESEM models should, theoretically, provide a better representation of the data. For other studies, the relationships between exo- and endogenous factors should be clearly articulated	Yes	Literature review
2	Plan for the most appropriate sample size	Determine and plan for the most appropriate sample size for the study. This can be done in many ways [c.f. (70, 72, 88)]. Monte Carlo simulations are preferred [c.f. (73)]	No	
***Data Preparation Phase***				
3	Data cleaning, screening, and preparation	Screen the data for potential issues (e.g. outliers), and prepare it for further analysis. Data quality checks should also be performed [c.f. (64)]	No	
		Decide upon an appropriate missing values strategy (e.g. Multiple imputations, FIML, sensitivity analysis)	Yes	Methods: statistical analysis
4	Determine the most appropriate software, estimation method, rotation and procedure for the analysis	Decide upon and report the software packages (and version number) that will be used for the analysis. ESEM is fully integrated in Mplus, but is currently only partially supported in R.	Yes	Methods: statistical analysis
		If data follows a multivariate normal distribution, employ the Maximum Likelihood Estimator in Mplus. If data is not normally distributed, either transform the data or use more robust estimation methods in Mplus (e.g. MLR, WLSMV). For all models with continuous indicators, the MLR estimator is also appropriate; models comprised of ordinal indicators WLSMV should be used.	Yes	Methods: statistical analysis
		Decide upon the most appropriate rotation method (Geomin / Target / Target Orthogonal). Geomin rotations (with an epsilon value of .5) for more exploratory approaches. Target rotations for confirmatory approaches. (Target) Orthogonal rotations are used for bifactor ESEM modeling.	Yes	Methods: statistical analysis
		Describe the analysis procedure to be employed		
***Data Analysis and Reporting Phase***				
5	Determine appropriate goodness-of-fit indices, and indicators of measurement quality	Decide upon which goodness-of-fit indices are most appropriate for the analyses (e.g., TLI/CFI, RMSEA, RMSEA Confidence Intervals etc). Report each index as well as the cut-off criteria to be considered. Multiple indicators are to be mentioned (c.f. Table 2). CFI/TLI/RMSEA should always be employed as the primary criterion.	Yes	Methods: statistical analysis
		Decide upon a priori indicators of measurement quality to be considered for the study (e.g. Standardized λ > 0.40; item uniqueness > 0.1 but <0.9; cross-loading tolerance levels; overall R2). However, the results should be considered in the context of the study and what they might mean or indicate without being rigid about minor deviations from the chosen guidelines.	Yes	Methods: statistical analysis
6	Estimate and report the model fit indicators for competing CFA Models	Multiple measurement models need to be estimated and their model fit statistics reported. Only models with theoretical justification should be estimated. The following models could be estimated: (1) Unidimensional model, (2) correlated first-order factorial models, (3) second-order or 'hierarchal' factorial model, and (4) bifactor models	Yes	Results: competing measurement models
		Report any potential modifications made to enhance model fit	Yes	Results: competing measurement models
		Tabulate all goodness-of-fit indices for all CFA models in a single table and indicate which models meet the pre-defined criteria mentioned and to make the comparison reader-friendly (refer to Point 5).	Yes	Results: competing measurement models
7	Estimate and report the model fit indicators for competing ESEM Models	Multiple ESEM measurement models should be estimated and model fit statistics reported. In principle, the ESEM alternatives to the traditional CFA models estimated in Point 6, should be reported. The following ESEM models could be estimated: (1) correlated first-order factorial ESEM models, (2) second-order or 'hierarchal' factorial ESEM model, (3) bifactor ESEM models and (4) ESEM within CFA models for use in structural models with other factors	Yes	Results: competing measurement models
		Tabulate all goodness-of-fit indices for all ESEM Models into the same table as the CFA models and indicate which models meet the pre-defined criteria mentioned in Point 5.	Yes	Results: competing measurement models
8	Compare CFA and ESEM models to determine the best fitting model for the data	CFA and ESEM models need to be compared against one another with the goodness-of-fit and measurement quality criteria mentioned in Point 5. Models that show comparatively better model fit should be retained for further analysis. It is, however, important to note that model fit should not be the only consideration, but the parameter estimates should also be closely inspected and considered.	Yes	Results: competing measurement models
		To retain ESEM models for further analysis, the following conditions need to be met:	Yes	Results: competing measurement models
		(a) The ESEM model should ideally show better data-model fit than the corresponding CFA model (including the same number of factors defined similarly). If the factor correlations for the ESEM model are smaller than those of the CFA model, then the ESEM model should be retained even if it fits as well as the CFA model.	Yes	Results: competing measurement models
		(b) For correlated factors models, the ESEM model should show reduced factor correlations	Yes	Results: competing measurement models
		(c) The ESEM model should only show small to medium cross-loadings. Should larger cross-loadings exist, then there should be a theoretical explanation presented for such. Perhaps there are ‘wording' effects or some logic that researchers can use to explain this.	Yes	Results: competing measurement models
		(d) The estimated latent factors within the ESEM model should be well defined	Yes	Results: competing measurement models
		(e) Should there be multiple medium to large cross-loadings in the ESEM model, it could indicate support for the presence of a larger global factor, and therefore the bifactor ESEM model could be explored.	Yes	Results: competing measurement models
		(f) Additional factors to consider for bifactor models: This model should ideally show better data-model fit than the corresponding CFA and ESEM models, there should be a well-defined G-Factor (where all items load significantly on such), and reasonably well defined S-Factors (cross- and non-significant loadings are permitted). For bifactor models, model fit should not be the only indicator informing a decision to retain. Researchers should also inspect parameter estimates before making final decisions.	Yes	Results: competing measurement models
9	Report factorial correlations	For the final retained measurement model (s), the factor correlations should be reported. This cannot be done for bifactor Models . Smaller factor correlations mean better discrimination between factors. The model with the smallest factor correlation is usually retained, however, decisions should be based in the context of the other considerations (model fit, measurement quality and parameter estimates) mentioned earlier.	Yes	Results: factorial correlations
10	Report and compare item level parameters and reliability	Item level parameters and indicators of measurement quality (standardized factor loadings, standard errors, item-level residual variances), as well as levels of reliability (composite reliability, or omega,), should be tabulated and reported.	Yes	Results: item level parameters
		Note, that if a bifactor CFA model is retained, editors or reviewers may request additional information such as the Explained Common Variance (ECV), the H-factor, the Factor Determinacy indicator, the Item level ECV, the Percent of Uncontaminated Correlations (PUC) and the Average Relative Bias Parameters could also be reported as additional indicators of reliability and measurement quality [For a tutorial cf. (84)].	Yes	Results: item level parameters
		Decide upon appropriate indicators of reliability for both the CFA and ESEM models, such as composite reliability [ρ > 0.80; (85)], or Mc Donald's Omega [ω > 0.70; (86)].		
		Report the level of reliability for each (sub) scale of the instrument	Yes	Results: item level parameters
***Further or Additional Analysis***				
11	For further or additional analysis, the best fitting ESEM model is respecified as a CFA model through the ESEM-within-CFA estimation procedure. This affords the opportunity to use the ESEM-within-CFA model for more complex estimation procedures such as invariance testing, multi-group analysis, latent growth models, structural models and the like.		Yes	Results

## References

[1] Donaldson SI Van Zyl LE Donaldson SI. PERMA+4: a framework for work-related wellbeing, performance and positive organizational psychology 2.0. Front Psychol. (2021) 13:1–13. 10.3389/fpsyg.2021.817244 PMC8819083 35140667

[2] Khademi R Najafi M. Tracing the historical roots of positive psychology by reference publication year spectroscopy (RPYS): a scientometrics perspective. Curr Psychol. (2020) 39:438–44. 10.1007/s12144-018-0044-z

[3] Martín-del-Río B Neipp MC García-Selva A Solanes-Puchol A. Positive organisational psychology: a bibliometric review and science mapping analysis. Int J Environ Res Public Health. (2021) 18:5222. 10.3390/ijerph18105222 34068995 PMC8157200

[4] Gallagher MW Lopez SJ. Positive Psychological Assessment: A Handbook of Models and Measures. Washington, DC: American Psychological Association (2002).

[5] Efendic E Van Zyl LE. On reproducibility and replicability: Arguing for open science practices and methodological improvements at the South African Journal of Industrial Psychology. SA J Ind Psychol. (2019) 45:a1607. 10.4102/sajip.v45i0.1607

[6] Earp BD Trafimow D. Replication, falsification, and the crisis of confidence in social psychology. Front Psychol. (2015) 6:621 10.3389/fpsyg.2015.00621 PMC4436798 26042061

[7] Van Zyl LE. Enhancing scientific credibility: an open science strategy for the South African Journal of Industrial Psychology. SA J Ind Psychol. (2019) 45:a1768. 10.4102/sajip.v45i0.1768

[8] Wong PTP Roy S. Critique of positive psychology and positive interventions. In: Brown NJL, Lomas T, Eiroa-Orosa FJ, editors. The Routledge International Handbook of Critical Positive Psychology. London: Routledge (2017). 10.4324/9781315659794-12

[9] Laher S. Psychological assessment in Africa: The time is now!. Afr J Psychol Assess. (2020) 1:1–3. 10.4102/ajopa.v1i0.1133240131

[10] Snow NE. Positive psychology, the classification of character strengths and virtues, and issues of measurement. J Posit Psychol. (2019) 14:20–31. 10.1080/17439760.2018.1528376

[11] Warren MA Donaldson SI. Scientific Advances in Positive Psychology. New York, NY: Praeger (2017).

[12] Brown NJL Sokal AD Friedman HL. The complex dynamics of wishful thinking: the critical positivity ratio. Am Psychol. (2013) 68:801–13. 10.1037/a0032850 23855896

[13] Brown NJL Sokal AD Friedman HL. Positive psychology and romantic scientism. Am Psychol. (2014) 69:636–7. 10.1037/a0037390 25197852

[14] Keyes CLM. Mental illness and/or mental health? investigating axioms of the complete state model of health. J Consult Clin Psychol. (2005) 73:539–48. 10.1037/0022-006X.73.3.539 15982151

[15] Jovanović V. Structural validity of the Mental Health Continuum-Short Form: the bifactor model of emotional, social and psychological wellbeing. Pers Ind Diff . (2015) 75:154–9. 10.1016/j.paid.2014.11.026

[16] Keyes CL Wissing M Potgieter JP Temane M Kruger A Van Rooy S. Evaluation of the mental health continuum–short form (MHC–SF) in setswana-speaking South Africans. Clin Psychol Psychother. (2008) 15:181–92. 10.1002/cpp.572 19115439

[17] Lamers SM Westerhof GJ Bohlmeijer ET ten Klooster PM Keyes CL. Evaluating the psychometric properties of the mental health continuum-short form (MHC-SF). J Clin Psychol. (2011) 67:99–110. 10.1002/jclp.20741 20973032

[18] Zemojtel-Piotrowska M Piotrowski JP Osin EN Cieciuch J Adams BG Ardi R . The mental health continuum-short form: The structure and application for cross-cultural studies–A 38 nation study. J Clin Psychol. (2018) 74:1034–52. 10.1002/jclp.22570 29380877

[19] Keyes CLM Shmotkin D Ryff CD. Optimizing well-being: The empirical encounter of two traditions. J Pers Soc Psychol. (2002) 82:1007–22. 10.1037/0022-3514.82.6.1007 12051575

[20] Morin AJS Myers ND Lee S. Modern factor analytic techniques: bifactor models, exploratory Exploratory Structural Equation Modeling (ESEM),19 structural equation modeling and bifactor-ESEM. In: Tenenbaum G, Eklund RC, editors. Handbook of Sport Psychology, 4th Edition, Vol. 2. New York, NY: Wiley Publishers (2020). p. 1044–73. 10.1002/9781119568124.ch51

[21] Marsh HW Muthén B Asparouhov T Lüdtke O Robitzsch A Morin AJ . Exploratory structural equation modeling, integrating CFA and EFA: application to students' evaluations of university teaching. Struct Equ Model. (2009) 16:439–76. 10.1080/10705510903008220

[22] Marsh HW Morin AJ Parker PD Kaur G. Exploratory structural equation modeling: An integration of the best features of exploratory and confirmatory factor analysis. Annu Rev Clin Psychol. (2014) 10:85–110. 10.1146/annurev-clinpsy-032813-153700 24313568

[23] Brown T. A. (2015). Confirmatory factor analysis for applied research (2nd ed.). New York, NY: Guilford Press.

[24] Morin AJS Arens A Marsh H. A bifactor exploratory structural equation modeling framework for the identification of distinct sources of construct-relevant psychometric multidimensionality. Struct Equ Model. (2016) 23:116–39. 10.1080/10705511.2014.961800

[25] Joshanloo M Jovanović V. The factor structure of the mental health continuum-short form (MHC-SF) in Serbia: an evaluation using exploratory structural equation modeling. J Ment Health. (2017) 26:510–5. 10.1080/09638237.2016.1222058 27690711

[26] Joreskog KG. A general approach to confirmatory maximum likelihood factor analysis. Psychometrika. (1969) 34:183–202. 10.1007/BF02289343

[27] Cao C Liang X. Sensitivity of fit measures to lack of measurement invariance in exploratory structural equation modeling. Struct Equat Model. (2021) 2021:1–11. 10.1080/10705511.2021.1975287

[28] Marsh HW. Application of confirmatory factor analysis and structural equation modeling insport/exercise psychology. In: Tenenbaum G, Eklund RC, editors. Handbook of Sport Psychology (3rd Ed). New York, NY: Wiley Publishers. (2007). p. 774–98. 10.1002/9781118270011.ch35

[29] Xiao Y Liu H Hau K-T. A comparison of CFA, ESEM, and BSEM in test structure analysis. Struct Equ Model. (2019) 26:665–77. 10.1080/10705511.2018.1562928

[30] Asparouhov T Muthén B. Exploratory structural equation modeling. Struct Equat Model. (2009) 16:397–438. 10.1080/10705510903008204

[31] Mai Y Zhang Z Wen Z. Comparing exploratory structural equation modeling and existing approaches for multiple regression with latent variables. Struct Equat Model. (2018) 25:737–49. 10.1080/10705511.2018.1444993

[32] Hu LT Bentler PM. Cutoff criteria for fit indexes in covariance structure analysis: conventional criteria versus new alternatives. Struct Equ Model. (1999) 6:1–55. 10.1080/10705519909540118

[33] McNeish D An J Hancock GR. The thorny relation between measurement quality and fit index cut-offs in latent variable models. J Pers Assess. (2018) 100:43–52. 10.1080/00223891.2017.1281286 28631976

[34] McNeish D Hancock GR. The effect of measurement quality on targeted structural model fit indices: a comment on Lance, Beck, Fan, and Carter (2016). Psychol Methods. (2018) 23:184–90. 10.1037/met0000157 29517268

[35] Shi D Lee T Maydeu-Olivares A. Understanding the model size effect on SEM fit indices. Educ Psychol Meas. (2019) 79:310–34. 10.1177/0013164418783530 30911195 PMC6425088

[36] Gucciardi DF Zyphur MJ. Exploratory structural equation modelling and Bayesian estimation. In: Ntoumanis N, Myers ND, editors. An Introduction to Intermediate and Advanced Statistical Analyses for Sport and Exercise Scientists. New Jersey, NY: Wiley (2016). p. 172–94.

[37] Morin AJS. Exploratory structural equation modeling. In: Hoyle RH, editor. Handbook of Structural Equation Modeling, Second Edition. New York, NY. Guilford Press (2021).

[38] Van Zyl LE Heijenk B Klibert J Shankland R Verger NB Rothmann S . Grit across nations: an investigation into the cross-national equivalence of the Grit-O Scale. J Happiness Stud. (2021).

[39] Marsh HW Guo J Dicke T Parker PD Craven RG. Confirmatory factor analysis (CFA), exploratory structural equation modeling (ESEM), and set-ESEM: optimal balance between goodness of fit and parsimony. Multivariate Behav Res. (2020) 55:102–19. 10.1080/00273171.2019.1602503 31204844

[40] Tóth-Király I Morin AJ Gillet N Bothe B Nadon L Rigó A . Refining the assessment of need supportive and need thwarting interpersonal behaviors using the bifactor exploratory structural equation modeling framework. Curr Psychol. (2020) 2020:1–15. 10.1007/s12144-020-00828-8

[41] Tóth-Király I Bõthe B Rigó A Orosz G. An illustration of the exploratory structural equation modeling (ESEM) framework on the passion scale. Front Psychol. (2017) 8:1968. 10.3389/fpsyg.2017.01968 29163325 PMC5681952

[42] Morin AJS Marsh HW Nagengast B. Chapter 10. exploratory structural equation modeling. In: Hancock GR, Mueller RO, editors. Structural Equation Modeling: A Second Course (2nd ed.). Charlotte, NC: Information Age Publishing, Inc. (2013).

[43] Lovibond SH Lovibond PF. Manual for the Depression Anxiety Stress Scales (2nd. Ed.). Sydney, NSW: Psychology Foundation (1995). 10.1037/t01004-000

[44] Morin AJS Asparouhov T. Estimation of a Hierarchical Exploratory Structural Equation Model (ESEM) Using ESEM-Within-CFA. Montreal, QC: Substantive Methodological Synergy Research Laboratory (2018).

[45] Howard JL Gagné M Morin AJ Forest J. Using bifactor exploratory structural equation modeling to test for a continuum structure of motivation. J Manag. (2018) 44:2638–64. 10.1177/014920631664565330909847

[46] Longo Y Jovanović V Sampaio de Carvalho J Karaś D. The general factor of wellbeing: multinational evidence using bifactor ESEM on the mental health continuum–short form. Assessment. (2020) 27:596–606. 10.1177/1073191117748394 29281897

[47] Rogoza R Truong Thi KH Rózycka-Tran J Piotrowski J Zemojtel-Piotrowska M. Psychometric properties of the MHC-SF: an integration of the existing measurement approaches. J Clin Psychol. (2018) 74:1742–58. 10.1002/jclp.22626 29687455

[48] Sànchez-Oliva D Morin AJS Teixeira PJ Carraça EV Palmeira AL Silva MN. A bifactor-exploratory structural equation modelling representation of the structure of basic psychological needs at work scale. J Vocat Behav. (2017) 98:173–87. 10.1016/j.jvb.2016.12.001

[49] Van Zyl LE Olckers C Roll LC. The psychometric properties of the Grit-O scale within the Twente region in Netherlands: An ICM-CFA vs. ESEM approach. Front Psychol. (2020) 11:796. 10.3389/fpsyg.2020.00796 32457679 PMC7223155

[50] Muthén LK Muthén BO. Mplus (Version 8.6) [Statistical software]. Los Angeles, CA: Muthén and Muthén (2021).

[51] Van Zyl LE Rothmann S. Evidence-Based Positive Psychological Intervention Practices in Multicultural Contexts. Cham: Springer (2019). 10.1007/978-3-030-20311-5

[52] Van Zyl LE Roll LC Stander MW Richter S. Positive psychological coaching definitions and models: a systematic literature review. Front Psychol. (2020) 11:793. 10.3389/fpsyg.2020.00793 32435218 PMC7218139

[53] World Health Organization. Promoting Mental Health: Concepts, Emerging Evidence, Practice: Summary Report. Geneva: World Health Organization (2004).

[54] Westerhof GJ Keyes CL. Mental illness and mental health: The two continua model across the lifespan. J Adult Dev. (2010) 17:110–9. 10.1007/s10804-009-9082-y 20502508 PMC2866965

[55] Keyes CL. The mental health continuum: from languishing to flourishing in life. J Health Soc Behav. (2002) 43:207–22. 12096700

[56] Keyes CL Yao J Hybels CF Milstein G Proeschold-Bell RJ. Are changes in positive mental health associated with increased likelihood of depression over a two year period? A test of the mental health promotion and protection hypotheses. J Affect Disord. (2020) 270:136–42. 32339105 10.1016/j.jad.2020.03.056

[57] Richter S Van Zyl LE Roll LC Stander MW. Positive psychological coaching tools: a systematic literature review. Front Psychiatry. (2021) 12:667200. 10.3389/fpsyt.2021.667200 34305674 PMC8298836

[58] Van Zyl LE Olckers C. The mental health continuum-short form in organisational contexts: factorial validity, invariance, and internal consistency. Eur J Ment Health. (2019) 14:230–59. 10.5708/EJMH.14.2019.2.2

[59] Keyes CL Grzywacz JG. Health as a complete state: the added value in work performance and healthcare costs. J Occup Environ Med. (2005) 47:523–32. 10.1097/01.jom.0000161737.21198.3a 15891532

[60] Gignac GE. The higher-order model imposes a proportionality constraint: That is why the bifactor model tends to fit better. Intell. (2016) 55:57–68. 10.1016/j.intell.2016.01.006

[61] Lamborn P Cramer KM Riberdy A. The structural validity and measurement invariance of the mental health continuum-short form (MHC-SF) in a large Canadian sample. J Well-Being Asses. (2016) 2:1–19.

[62] Joshanloo M Lamers SM. Reinvestigation of the factor structure of the MHC-SF in the Netherlands: contributions of exploratory structural equation modeling. Pers Individ Differ. (2018) 97:8–12. 10.1016/j.paid.2016.02.089

[63] De Beer LT Van Zyl LE. ESEM Code Generator for Mplus. (2019). Retrieved from: https://www.surveyhost.co.za/esem/ (accessed December 8, 2021).

[64] Buchanan EM Scofield JE. Methods to detect low quality data and its implication for psychological research. Behav Res Methods. (2018) 50:2586–96. 10.3758/s13428-018-1035-6 29542063

[65] De Beurs E Zitman F. De Brief Symptom Inventory (BSI): De betrouwbaarheid en validiteit van een handzaam alternatief. Maandblad Geestelijke Volksgezondheid. (2006) 61:120–41.

[66] Morrison TG Morrison MA McCutcheon JM. Best practice recommendations for using structural equation modelling in psychological research. Psychol. (2017) 8:1326–41. 10.4236/psych.2017.8908628233581

[67] Guo J Marsh HW Parker PD Dicke T Lüdtke O Diallo TM. A systematic evaluation and comparison between exploratory structural equation modeling and Bayesian structural equation modeling. Struct Equat Model. (2019) 26:529–56. 10.1080/10705511.2018.1554999

[68] Kyriazos TA. Applied psychometrics: sample size and sample power considerations in factor analysis (EFA, CFA) and SEM in general. Psychology. (2018) 9:2207–30. 10.4236/psych.2018.98126

[69] Myers ND Ntoumanis N Gunnell KE Gucciardi DF Lee S. A review of some emergent quantitative analyses in sport and exercise psychology. Int Rev Sport Exerc Psychol. (2018) 11:70–100. 10.1080/1750984X.2017.1317356

[70] Hancock GR Freeman MJ. Power and sample size for the root mean square error of approximation test of not close fit in structural equation modeling. Educ. Psychol Measur. (2016) 61:741–58. 10.1177/00131640121971491

[71] MacCallum RC Browne MW Sugawara HM. Power analysis and determination of sample size for covariance structure modeling. Psychol Methods. (1996) 1:130–49. 10.1037/1082-989X.1.2.130

[72] Maxwell SE Kelley K Rausch JR. Sample size planning for statistical power and accuracy in parameter estimation. Ann Rev Psychol. (2007) 59:537–63. 10.1146/annurev.psych.59.103006.093735 17937603

[73] Wolf EJ Harrington KM Clark SL Miller MW. Sample size requirements for structural equation models: An evaluation of power, bias, and solution propriety. Educ Psychol Meas. (2013) 73:913–34. 10.1177/0013164413495237 25705052 PMC4334479

[74] Satorra A Saris WE. Power of the likelihood ratio test in covariance structure analysis. Psychometrika. (1985) 50:83–90. 10.1007/BF0229415011393898

[75] Muthén LK Muthén BO. How to use a Monte Carlo study to decide on sample size and determine power. Struct Equ Model. (2002) 9:599–620. 10.1207/S15328007SEM0904_8

[76] Hair J Jr Black W Babin B Anderson R. Multivariate Data Analysis (7th ed.). London: Pearson New International Edition (2014).

[77] Kline RB. Principles and Practice of Structural Equation Modeling. 4th ed. New York, NY: Guilford publications (2015). p. 96–106.

[78] Shi D Maydeu-Olivares A Rosseel Y. Assessing fit in ordinal factor analysis models: SRMR vs. RMSEA Struct Equat Model. (2020) 27:1–15. 10.1080/10705511.2019.1611434

[79] Brown TA. Confirmatory Factor Analysis For Applied Researchers. New York, NY: Guilford publications (2006).

[80] Chen FF. Sensitivity of goodness of fit indexes to lack of measurement invariance. Struct Equ Model. (2017) 14:464–504. 10.1080/10705510701301834

[81] Wong J Wong X. (2020). Structural Equation Modelling: Applications Using Mplus, 2nd ed. Chichester: Wiley & Sons. p. 536.

[82] Murray AL Johnson W. The limitations of model fit in comparing the bifactor versus higher-order models of human cognitive ability structure. Intell. (2013) 41:407–22. 10.1016/j.intell.2013.06.004

[83] Zijlmans EA Tijmstra J van der Ark LA Sijtsma K. Item-score reliability as a selection tool in test construction. Front Psychol. 9:2298. 10.3389/fpsyg.2018.02298 PMC6336834 30687144

[84] Dueber DM,. Bifactor Indices Calculator: A Microsoft Excel-Based Tool to Calculate Various Indices Relevant to Bifactor CFA Models. (2017). Available online at: http://sites.education.uky.edu/apslab/resources/ (accessed December 8, 2021).

[85] Raykov T. Evaluation of scale reliability for unidimensional measures using latent variable modeling. Meas Eval Couns Dev. (2009) 42:223–32. 10.1177/0748175609344096

[86] Hayes AF Coutts JJ. Use omega rather than Cronbach's alpha for estimating reliability. Commun Methods Measures. (2020) 14:1–24. 10.1080/19312458.2020.1718629

[87] Morin AJS. Exploratory structural equation modeling. In: Hoyle RH, editor. Handbook of Structural Equation Modeling, 2nd ed. New York, NY: Guilford publications (In Press).

[88] Jak S Jorgensen TD Verdam MGE Oort FJ Elffers L. Analytical power calculations for structural equation modeling: a tutorial and Shiny app. Behav Res Methods. (2021) 53:1385–406. 10.3758/s13428-020-01479-0 33140375 PMC8367885

[89] Kim HY. Statistical notes for clinical researchers: assessing normal distribution (2) using skewness and kurtosis. Restor Dent Endod. (2013) 38:52–4. 10.5395/rde.2013.38.1.52 23495371 PMC3591587

[90] Disabato DJ Goodman FR Kashdan TB Short JL Jarden A. Different types of well-being? A cross-cultural examination of hedonic and eudaimonic well-being. Psychol Assess. (2016) 28:471–82. 10.1037/pas0000209 26348031

